# Towards personalized and optimized fitting of cochlear implants

**DOI:** 10.3389/fnins.2023.1183126

**Published:** 2023-07-13

**Authors:** A. John Van Opstal, Elisabeth Noordanus

**Affiliations:** Donders Centre for Neuroscience, Section Neurophysics, Radboud University, Nijmegen, Netherlands

**Keywords:** cochlear implant technology, objective measures, personalized health care, psychophysics, electrophysiology, reaction times

## Abstract

A cochlear implant (CI) is a neurotechnological device that restores total sensorineural hearing loss. It contains a sophisticated speech processor that analyzes and transforms the acoustic input. It distributes its time-enveloped spectral content to the auditory nerve as electrical pulsed stimulation trains of selected frequency channels on a multi-contact electrode that is surgically inserted in the cochlear duct. This remarkable brain interface enables the deaf to regain hearing and understand speech. However, tuning of the large (>50) number of parameters of the speech processor, so-called “device fitting,” is a tedious and complex process, which is mainly carried out in the clinic through ‘one-size-fits-all’ procedures. Current fitting typically relies on limited and often subjective data that must be collected in limited time. Despite the success of the CI as a hearing-restoration device, variability in speech-recognition scores among users is still very large, and mostly unexplained. The major factors that underly this variability incorporate three levels: (i) variability in auditory-system *malfunction* of CI-users, (ii) variability in the *selectivity* of electrode-to-auditory nerve (EL-AN) activation, and (iii) lack of objective *perceptual* measures to optimize the fitting. We argue that variability in speech recognition can only be alleviated by using objective patient-specific data for an individualized fitting procedure, which incorporates knowledge from all three levels. In this paper, we propose a series of experiments, aimed at collecting a large amount of objective (i.e., quantitative, reproducible, and reliable) data that characterize the three processing levels of the user’s auditory system. Machine-learning algorithms that process these data will eventually enable the clinician to derive reliable and personalized characteristics of the user’s auditory system, the quality of EL-AN signal transfer, and predictions of the perceptual effects of changes in the current fitting.

## 1. Introduction

According to the WHO (The World Health Organization, 2021), over 5% of the world’s population currently require rehabilitation to address their disabling hearing loss. This concerns 432 million adults and 34 million children. Because of the increasing proportion of elderly in the world’s population, it is estimated that by 2050 over 700 million people – nearly 10% – will have a disabling hearing loss. The estimated incidence of sensory-neural deafness [i.e., in need of a cochlear implant (CI)] amounts to ~0.3% of the population, which concerns about 600,000 individuals in the EU and USA, and 21 million persons worldwide. Consequently, an estimated US$ 1 trillion is lost each year because hearing loss for the population at large is not adequately addressed.

Without treatment, hearing loss leads to social isolation, significant delays in intellectual development, and degraded communicative skills. Moreover, adequate *binaural hearing* is crucial to safely navigate in unpredictable urban environments (Avan et al., 2015; Hoppe et al., 2018; Rak et al., 2019; Sivonen et al., 2021), as the ability to localize sounds prevents life-threatening situations in traffic and improves source separation and speech understanding in noisy scenes like in a crowd (King et al., 2021).

Clearly, hearing loss (HL) poses a huge problem. Hearing-aid manufacturers and researchers search for methods that improve predictability and reduce variability of success performance rates after implantation, and to optimize binaural hearing performance after bilateral implantation. Current CI technologies for the sensory-neural deaf (those suffering from >80 dB HL), and hearing-aids (HA) for the hearing-impaired (with conductive and mild sensory-neural loss; 40–80 dB HL) enable (partial) restoration and improvement of hearing and speech understanding, even when applied unilaterally (Wilson et al., 1991; Sousa et al., 2018). Binaural performance can in principle be enhanced with bilateral restoration (CI-CI, or HA-HA; Hoppe et al., 2018; King et al., 2021).

However, the CI-CI solution is not yet available in many countries for lack of objective evidence of a significant benefit of a second implant. Often, some residual (low-frequency) hearing remains in the ear contralateral to the CI, in which case a *bimodal* solution (HA-CI, i.e., acoustic-electric) aims to partially restore binaural hearing (Veugen et al., 2016, 2017; Hoppe et al., 2018; King et al., 2021; Van der Heijdt et al., in prep.). Yet, so far, bimodal performance results indicate that true binaural integration is seriously hampered because of the vastly different stimulation modes of the auditory nerves by either device: acoustic broadband, spectrally continuous stimulation by the conventional HA vs. electric pulsatile stimulation of a low number of electrode channels (typically 16–20) by the CI. This profound mismatch seriously challenges central binaural integration, as well as inter-device communication algorithms (Veugen et al., 2016, 2017; Van der Heijdt et al., in prep.). Note that the latter also holds for current bilateral HA-HA and CI-CI solutions (Veugen et al., 2016; Ausili et al., 2020).

### 1.1. Clinical challenges

**Figure 1:**
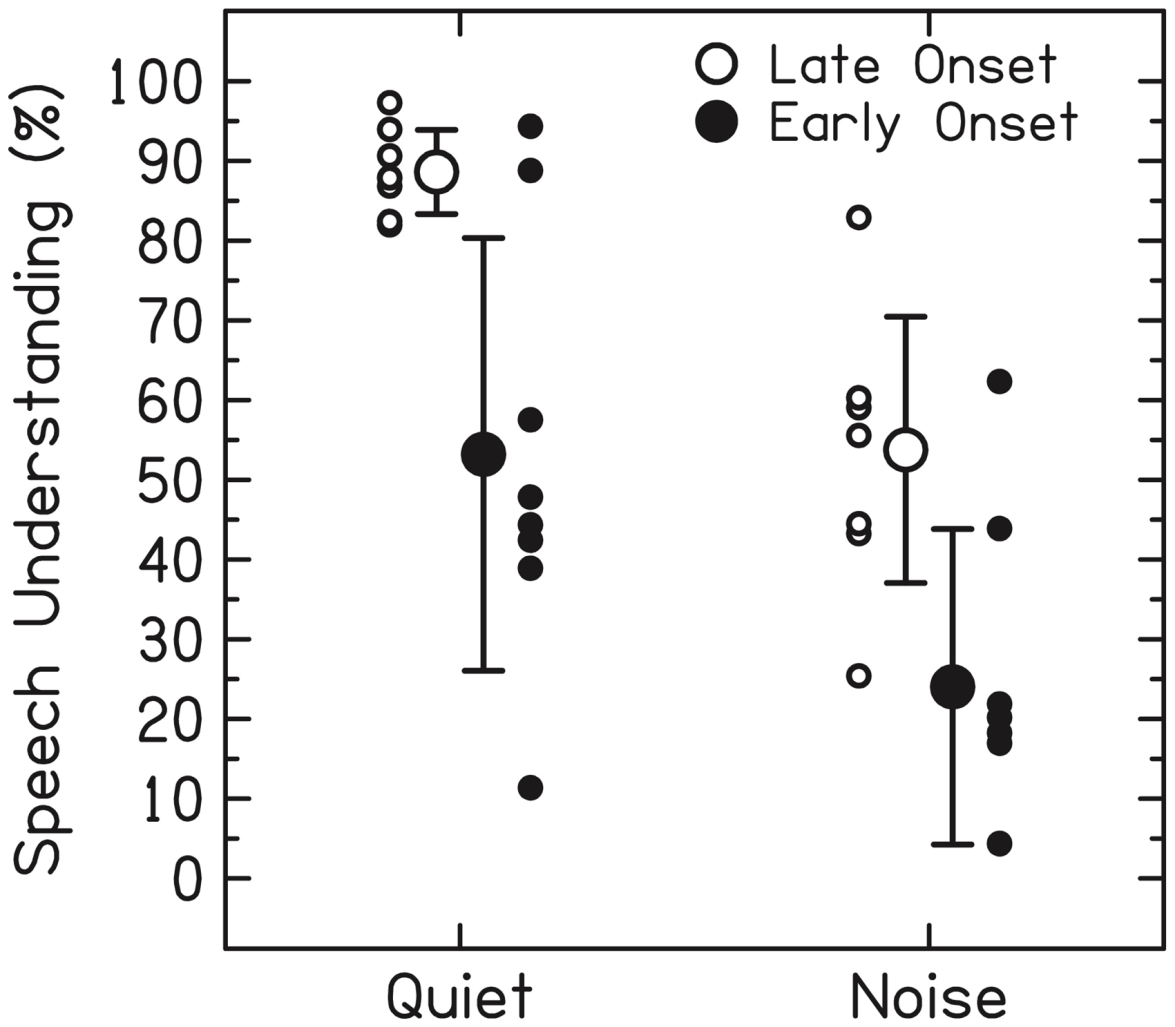
Speech understanding in quiet with a unilateral CI can be excellent (>80% correct) for late-onset (post-lingual) deaf listeners (open dots, left), but there is huge variability for the early-onset (pre-lingual) deaf (solid dots; 10–95% correct scores). Both groups, however, show large variability and much lower success scores, for speech intelligibility in ambient noise. Data from two groups of eight age-matched unilateral CI-users (mean age: 57 years). All received the CI at an adult age. The noise was multi-talker babbling noise at 10 dB SNR. Large open/solid dots indicate the means with 1 SD [courtesy: acoustics today (Goupell, 2015), with permission].

It is widely acknowledged that adequate tuning of a hearing device (so-called ‘*fitting*’) should be based on solid objective auditory neural encoding and perceptual sensitivity measures (McKay et al., 2013; Büchner et al., 2015; De Vos et al., 2018; Canfarotta et al., 2020; Crowson et al., 2020; Canfarotta et al., 2021; Carlyon and Goehring, 2021), which up to now are rarely available in clinical practice. Most clinical tests are taken with subjective, self-reporting, responses from the participant, leading to unreliable, and irreproducible measures on which to base the fitting. As a result, most current fitting procedures follow a ‘*one-size-fits-all*’ strategy. Although, fortunately, this leads to acceptable auditory reception levels in many cases, there is still considerable and unexplained variability of actual auditory performance in CI recipients (Figure 1). This problem becomes especially prominent in their more challenging daily environment (Blamey et al., 2013; Pisoni et al., 2017; Tamati et al., 2020; Carlyon and Goehring, 2021; Dornhoffer et al., 2021; Heutink et al., 2021; Wathour et al., 2021). It is therefore thought that current fitting procedures are at best suboptimal and should be fundamentally improved. To achieve this, three important challenges must be tackled that are addressed in this paper:

**(i) What causes the variability in auditory performance among CI-recipients and bimodal (CI-HA) patients?** Figure 1 illustrates the outcome-variability problem for speech understanding in quiet and in noise for two age-matched groups with different etiologies (Blamey et al., 2013; Goupell, 2015; Heutink et al., 2021; Shafieibavani et al., 2021). Although the late-onset (i.e., acquired, post-lingual) deaf can reach high speech-recognition scores (>80%) in a quiet lab environment, the variability found in early-onset (pre-lingual) users is tremendous (10–90%). However, in a noisy setting, both groups yield highly variable performance scores. Similar variability has been reported for pitch perception (e.g., music appreciation, distinguishing male–female voices, recognizing emotional intonations), and for binaural-dependent measures like sound-localization performance of bimodal (CI-HA) and bilateral (CI-CI) users. In section 1.3.1 we will briefly outline some of the major factors underlying this variability.

**(ii) The number of device parameters to be adjusted is vast:** a single CI typically has more than 50 relevant tuneable parameters. Thus, finding an optimal personalized fitting is a daunting challenge that cannot be reliably tackled by hand, or by adopting a fixed set of values as in current clinical practice (Carlyon and Goehring, 2021). Section 1.3.2 will address this point.

**(iii) Binaural hearing requires successful neural integration of acoustic-electric information from either ear** to yield performance levels that exceed the simple sum from the individual devices (“1 + 1 > 2”). For example, sound localization in the horizontal plane requires a precise neural comparison of subtle interaural timing (ITD) and level (ILD) differences in the acoustic inputs (Agterberg et al., 2011, 2012; Van Opstal, 2016; Hoppe et al., 2018; Ausili et al., 2020). So far, such integration is rarely achieved in bimodal hearing, and it also shows considerable variability for bilateral users. Often, bilateral use of devices may even *hamper* performance because of inconsistent binaural signals. In such cases, bilateral stimulation might even induce the percept of disconnected sources at either ear, instead of a unified binaural auditory object. The important question is how bilaterally applied hearing devices (CI-CI, CI-HA, or HA-HA) can be best combined to restore and optimize binaural hearing of the hearing-impaired listener (Pisoni et al., 2017; Rak et al., 2019). We will further address this point in Section 1.3.3.

### 1.2. Technical challenges

Hearing-aid manufacturers work hard to develop combined bimodal (CI-HA) and bilateral (CI-CI) hearing solutions, by tackling the technical challenges that let devices communicate with each other with sufficient spectral-temporal resolution and binaural overlap, more precise (sub-millisecond) cross-device synchronization (Ausili et al., 2020) and providing an optimized dynamic range of the device signals to the impaired auditory system (Veugen et al., 2016, 2017). Yet, even if technical distortions at the input are resolved (Van der Heijdt et al., in prep.), the abovementioned challenges must be resolved *closed loop,* i.e., at the level of the listener’s *output*: the auditory perceptual performance, and neural response patterns. Ideally, these should be assessed with objective and reproducible measurements, instead of subjective verbal scores (Büchner et al., 2015; Crowson et al., 2020; Carlyon and Goehring, 2021). In the Proposed Methodology section, we specify several such experimental paradigms, some of which are standard practice, while others are novel proposals.

### 1.3. Underlying mechanisms and factors

#### 1.3.1. Variability in auditory performance

Much of the variability in CI performance outcomes (as in Figure 1) is due to a combination of several underlying factors (Blamey et al., 2013; Dornhoffer et al., 2021; Heutink et al., 2021). These include device issues like sub-optimal fitting of the many device parameters to the specific neurophysiological needs of the user (Van der Marel et al., 2015; Padilla and Landsberger, 2016; Canfarotta et al., 2020, 2021; Skidmore et al., 2021; Lo et al., 2022), neural factors like unknown differences in neural development and neural communication pathways between users (Palmer and Summerfield, 2002; Schwarz and Taylor, 2005; Picton, 2011), the etiology of the hearing loss, e.g., genetic factors, acoustic trauma, ototoxic drugs, etc., the age at implantation being pre-lingual or post-lingual, the duration of deafness, and cognitive factors like language comprehension or cognitive skills (Blamey et al., 2013; Dornhoffer et al., 2021; Heutink et al., 2021). To successfully address this problem requires better understanding of bottom-up and top-down acoustic-neural processing of the individual listener. Such knowledge would enable the clinician to provide informed fitting advice from objective information and to predict potential outcomes in auditory performance.

Currently, an acute measurement of speech comprehension taken after fitting (as often done in clinical practice) ignores the time needed for the user’s auditory system to adapt to the new settings. Improvements or success of the fitting is then monitored at successive follow-up visits to the clinic, e.g., at 2 weeks, 3, and 6 months post-implantation. Moreover, speech-comprehension tests may suffer from serious cognitive confounds. Clinical tests aim to reduce such cognitive influences by measuring, e.g., phoneme recognition scores. Yet, someone who hears perfectly well, but does not understand the task or the language, will yield low speech-recognition performance scores. Furthermore, speech is more than just an acoustic string of phonemes, as much of the comprehension also relies on non-acoustic factors, like prediction, expectation, context, and filling in. Therefore, a more objective assessment of auditory performance should rely on stimuli that are *a valid proxy* for (dynamic) speech, but do not suffer from these cognitive confounds.

#### 1.3.2. Optimizing the device fitting

The number of parameters that can be adjusted in a hearing device is typically vast. For example, a typical CI, like the Advanced Bionics HiRes Ultra 3D implant with Naída sound processor, or the Cochlear Nucleus implant with its Nucleus 8 processor, potentially has about 20–22 active frequency channels, in which each channel has multiple relevant tuneable parameters [e.g., minimum/maximumstimulation levels, attack and release times, automatic gain control (Padilla and Landsberger, 2016; Veugen et al., 2016; Wang et al., 2021; Van der Heijdt et al., in prep.)]. Moreover, adequate spatial mapping of the CI frequency channels (i.e., the stimulus spectrum) onto the appropriate (tonotopic) auditory nerve-locations (i.e., the spectral representation in the central nervous system, CNS) is an inherent problem (Canfarotta et al., 2020; Yang et al., 2020; Van der Willigen et al., 2023). Finally, selecting CI channels that should be excluded because of inadequate percepts (possibly due to excessive current spread, or to cross-electrode leakage) is a difficult task. Finding optimal settings for all these parameters is a daunting challenge that cannot be tackled by hand (as is currently done). Instead, it is understood more and more that statistical methods (e.g., based on machine-learning, or deep-learning neural nets) are required to address this fundamental problem (McKay et al., 2013; Büchner et al., 2015; Noorani and Carpenter, 2016; Sousa et al., 2018; Crowson et al., 2020; Carlyon and Goehring, 2021).

However, regardless the sophistication of data analysis and machine-learning methods, their success fully depends on the quality of their input data. In other words, ‘*garbage in, garbage out*’. Furthermore, such methods typically require vast amounts of training data to generate evermore reliable estimates. Studying these problems on small groups of patients with limited data sets will therefore not suffice to reveal the complexity of the many interacting factors mentioned above (Wathour et al., 2021).

We here argue that data collection should (a) be obtained from uniform procedures, based on scientifically sound paradigms that yield reproducible (objective) results from various stages in the auditory processing chain, and (b) combine the results from many [estimated at about 200 (Shafieibavani et al., 2021)] listeners over time to yield a large cumulative data base that would be applicable for each patient. This enables the field to build crucial fundamental knowledge about the detailed relationships between device settings and the resulting perceptual and auditory system performance measures of CI users.

#### 1.3.3. Bilateral or bimodal (CI-CI or CI-HA) device fitting

In bilateral restorative hearing, all potential factors described above for a single device also apply to each of the pair. However, on top of that, variability in success rates may also be (partly) attributed to inadequate communication between devices (Veugen et al., 2016, 2017; Ausili et al., 2020; Van der Heijdt et al., in prep.). The latter leads to a mismatch between binaurally applied input signals, which seriously distorts acoustically valid binaural cue information for adequate and unambiguous binaural integration. Binaural difference ITD and ILD cues are needed by the brain for sound-source localization in the horizontal plane (Van Opstal, 2016). Additional acoustic (e.g., spectral-temporal) and non-acoustic cues (e.g., vision, source familiarity) are required for sound-source segregation in complex acoustic scenes to facilitate speech comprehension, like at a cocktail party, or in a busy urban environment (Avan et al., 2015; Rak et al., 2019), and for the binaural binding of auditory events. These cues are quite subtle. For example, physical ITDs for frequencies up to about 1.5 kHz vary between ±0.6 ms from far-left to far-right locations, which requires sub-millisecond timing resolution (Van Opstal, 2016). This necessitates fine-structure time analysis of the inputs which is rarely available in current CI encoding strategies (Ausili et al., 2020). Similarly, ILDs vary from ±1 dB (2 kHz) to ±10 dB (>6 kHz; Van Wanrooij and Van Opstal, 2004; Van Opstal, 2016). Their extraction requires precise loudness balancing between devices (Veugen et al., 2016), which is potentially destroyed by automatic gain control in the processors (Van der Heijdt et al., in prep.). Because of the limited dynamic range and poor spectral-temporal resolution of the user (Zheng et al., 2017), this is a serious technical challenge for current devices. It is also unknown how much these requirements may be relaxed within these limitations to still allow for (near-)adequate binaural integration and hence to a potential bilateral benefit. Finally, reduction of binaural mismatch also requires matched frequency-allocation tables (FATs) of the devices (Van der Marel et al., 2015).

In our research, we have provided evidence that device synchronization and frequency matching can help to partially restore binaural integration, even when it is still far from optimal (Veugen et al., 2016; Ausili et al., 2020; Van der Heijdt et al., in prep.). At present, however, an optimal bilateral or bimodal solution does not exist.

### 1.4. Addressing the problem

In summary, the challenges described above require an interdisciplinary approach that contains the following elements:

1. Obtain better fundamental understanding of the CI-brain-response processing chain, by combining fundamental knowledge from electrophysiological measurements at the electrode-neuron interface, pre- and post-operative anatomical cochlear imaging measurements, neurobiological understanding of auditory nerve responses and the subsequent neural integration stages, with reliable psychophysical and perceptual response data from the listener. The approach will apply to unilateral CI users, to bimodal CI-HA recipients, and to bilateral CI-CI users.
2. Collect multimodal objective measurements from many unilateral CI, bimodal CI-HA and bilateral CI-CI users, all subjected to the *same* experimental paradigms (pre- and post-op), and construct a large cumulative, standardized, data base of device settings (‘*fitting*’), relevant patient characteristics, basic neurophysiological parameters, and auditory performance scores collected over time. These performance measures should be obtained from both the peripheral and central auditory system and from perceptual/behavioral response paradigms.
3. Obtain additional cumulative psychophysical data over time by measuring responses from patients in their home environment, e.g., by performing simple experiments with the help of an App.
4. Develop machine-learning algorithms that learn to predict the relationships between device settings, and changes therein, with the (multimodal) auditory performance scores. Ultimately, with such an algorithm (i.e., a listener-specific model), one hopes to do the reverse: find potential device settings that will likely lead to better perceptual performance scores.

## 2. Proposed methodology

**Figure 2:**
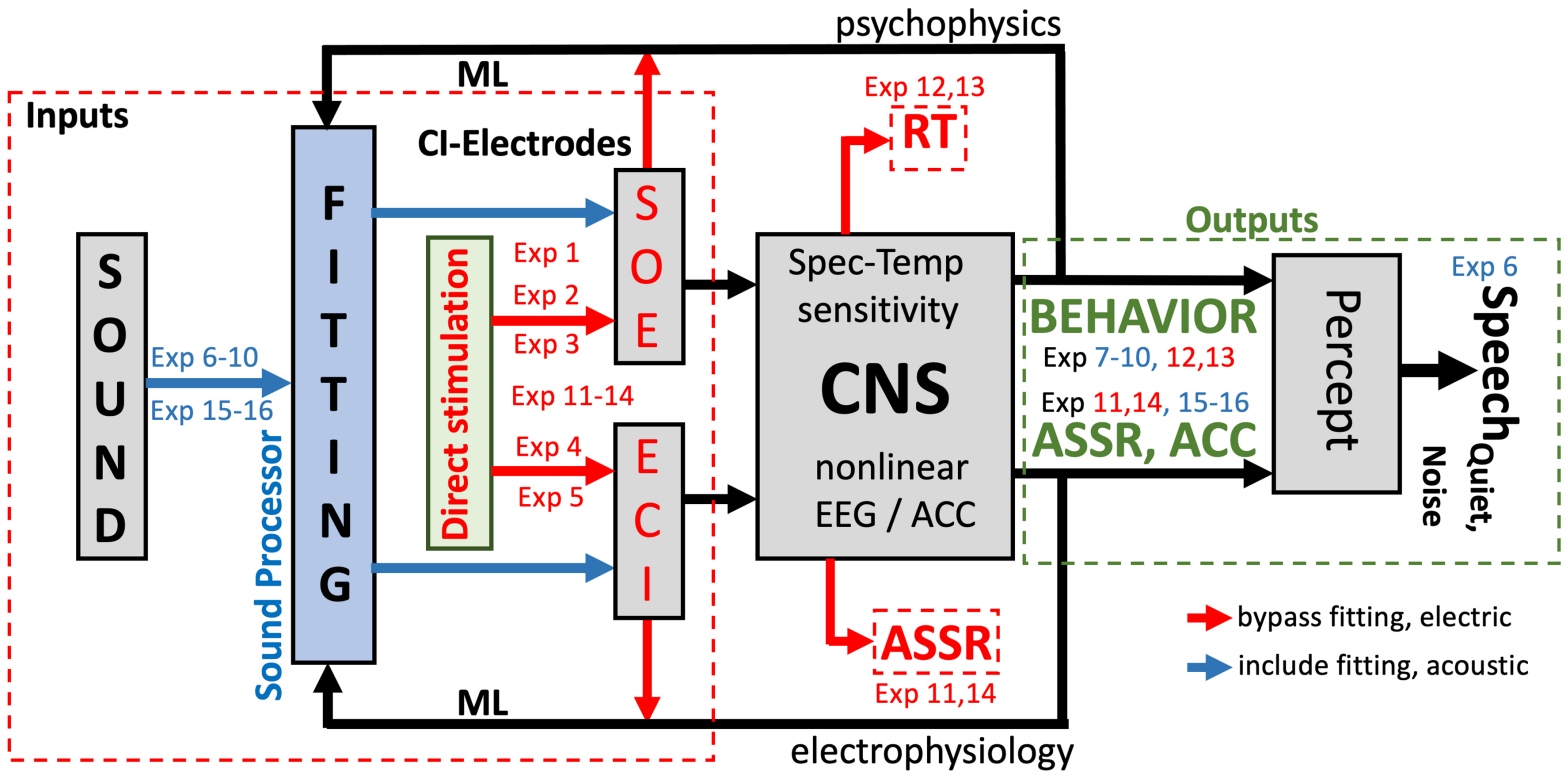
Signal transfer model for the auditory system of a unilateral CI user from sound (presented *via* the CI sound processor; blue arrows), or through direct stimulation of the electrodes (red arrows), to percept. Exp nrs. Refer to Table 1. ML: machine-learning algorithms that relate CI fitting to objective inputs (from SOE, ECI, eCAP, anatomy, direct stimulation; red) and multimodal output measures [reaction times (RT), psychometric data (behavior), and auditory steady-state responses in the EEG (ASSR) and an ACC in cortical evoked potentials]. The ML model predicts auditory percepts for the current fitting and proposes changes therein to optimize speech reception in quiet and in noise (Section 3.5). The model can be extended to promote optimal binaural integration for bilateral CI-CI and CI-HA hearing.

Table 1 proposes a series of auditory performance tests (Pisoni et al., 2017; Veugen, 2017; Wang et al., 2021; Veugen et al., 2022; Noordanus et al., in prep.) that allow for quantitative and reliable functional assessments of different aspects in the auditory processing chain. Together they will provide accurate objective insights into the capacities of the listener’s auditory system at different levels. Some of these experiments are carried out only once (i.e., prior to, or during surgery, or immediately post-op). Others are performed multiple times, either in the laboratory/clinical environment, or in the user’s own home environment. The tests are described in more detail below and are marked in Figure 2.

**Table 1:** Proposed set of experimental protocols to collect high-quality data from a large (*N* ~ 200) group of patients, taken from different clinical centers that all follow the same standardized procedures.

**Single-session assessment**		
Pre-operative intake tests	0.^*^	High-resolution MRI + CT scan of the cochlea; inspect the spiral ganglion. Binaural (un)aided audiograms; SRT_Q,N_ test; ST-ripple reaction-time test.
During surgery	1.	Electro-Cochleo-Graphy (EcochG) to assess residual haircell function.
Post-op anatomy	2.	CT scan of CI electrode position within the cochlear duct.
Electrophysiology	3.	Electrode cross-impedance (ECI) measurements along the CI array.
	4.	Spread of Auditory Nerve (AN) excitation (SOE) to single-electrode stimulation along the array.
	5.	eCAP to assess auditory nerve health. Stimulate different channels at different current strengths.
**Multiple-session assessments**		
*Psychophysics*		
	6.	Speech-reception performance in quiet and in noise with home-App.
	7.	Reaction times (RT) to spectrotemporal ripples with home-App.
	8.	Pitch and frequency discrimination thresholds with home-App.
	9.^‡^	Interaural time (ITD) and level (ILD) difference psychometrics.
	10.^*‡^	Free-field sound-localization (head orienting) in the horizontal plane.
	0b^*(‡)^	(Un)aided audiograms of either ear.
Direct CI electrode stimulation^*^		
	11.	Auditory steady-state responses (eASSRs) in the EEG for different pulse frequencies and pulse modulations on different contact pairs.
	12.	RT to sudden electrode change (e.g., from contacts 3 + 4 to 5 + 4; RT-eACC).
	13.	RT to a sudden modulation of electrode pulse-frequency.
	14.^‡^	Binaural eASSRs in the EEG.
	5b^*^	Repeat eCAP measurements (amplitude growth functions, thresholds).
*EEG*		
	15^*(‡)^	Monaural and (binaural) ASSRs in the EEG with acoustic tone complexes.
	16^*^	Acoustic change complex (ACC; averaged evoked potentials)

*Performed in the lab. ^‡^Only for bilateral (CI-CI or CI-HA) users. Experiments 0–5 are carried out, either prior to, or during, surgery, and post-op. Experiments 6–9 can be performed at home with the help of a dedicated smartphone App in multiple sessions. They can be spread over extended periods of time to assess neural plasticity, or long-term adaptation. Experiments 9–10 and 14 are only carried out in binaural (bilateral CI-CI, or CI-AH) listeners. All direct stimulation tests (11–14), free-field sound-localization experiments (10), audiograms (0b), and EEG recordings (15–16) are done in the lab. Note that direct stimulation paradigms circumvent the CI sound processor and fitting. See also Figure 2.

Note that not all Table 1 experiments can be performed on all CI-users. For example, very young CI recipients, say <2–3 years of age, or listeners with serious cognitive or motor deficits, may not comprehend the task needed to generate reliable psychophysical responses. Yet, even for those users, the combined set of electrophysiological and anatomical tests (experiments 0–5, 11, and 14–16) will yield valuable objective information for a better fitting, which may at a later stage be further improved by additional psychophysical data.

Figure 2 provides a schematic overview of the (single-sided) auditory processing chain that underlies the rationale of the proposed experiments (indicated by “*Exp nr*”), and the different levels in the auditory system that are targeted by the proposed experiments of Table 1. The main idea is to let the auditory brain process the pulsatile information from the CI in two different ways:

- Through the ‘normal’ acoustic-electric-neural pathway, which uses the full system, including the sound processor and its (updatable) algorithms (the blue arrows in Figure 2). ‘Fitting’ of the system and its effects on the listener follows this stimulation mode, and the psychophysical experiments 6–10 and EEG experiments 15 and 16 in Table 1 target this stimulation pathway.
- Through direct electrical stimulation of selected electrodes and the auditory pathways (red arrows in Figure 2), which circumvents the fitting and processing algorithms. The results from experiments 1–5 are used to map the signal transfer properties of the CI to the auditory nerve. Experiments 11–14 use this mode of stimulation to assess the direct electric-neural effects of pulsatile stimulation patterns on the listener’s auditory percept and on the EEG.

### 2.1. Rationale

The combined multimodal data generated by these protocols (anatomy, electrical auditory nerve measurements, audiograms, psychophysical responses, speech reception in quiet and in noise, and EEG data), in combination with the fitting parameters, will provide unique quantifiable information about the user’s auditory system at its different levels (given the current fitting). The ultimate challenge will be to identify the complex functional relationship(s) between the results from experiments 1–5, and 7–16 and the speech-reception performance of the listener in quiet and in noise of experiment 6. Note that any changes in the fitting will not affect the outcomes from experiments 1–5 and 11–14 but are expected to influence the measurements from the psychophysics, experiments 6–10, and the acoustic EEG results of experiments 15 and 16.

## 3. Experimental tests and pilot results

### 3.1. Single-session electrophysiology and anatomy

*Pre-operative clinical intake tests*. Prior to surgery, several standardized tests are to be performed to assess the auditory-perceptual performance levels of the future CI-user without the presence of an implant. These include a high-resolution anatomical MRI-CT scan to obtain a detailed image of the cochlear duct, the status of the spiral ganglion, and aided and unaided psychometric audiograms of either ear to measure potential low-frequency rest-hearing (experiment 0 in Table 1).

Finally, two psychophysical tests will be taken that determine the speech-reception thresholds in quiet and in noise (SRT_Q,N_) pre-op (for later comparisons with the implant), and reaction-time experiments for a selected set of spectrotemporal moving ripples that fall within the range of human speech modulations (these are described in Section 3.2.3).

*Post-operative clinical tests*. During surgery and post-operatively, four standard clinical tests should be performed to acquire valuable objective anatomical and electrophysiological data concerning the implant. Each of these tests establishes the *in-situ* properties of the electrode in the cochlea. During surgery, electrocochleography (ECochG) in experiment 1 can assess cochlear health (c.q., the presence of potential low-frequency residual hearing).

Second, a post-operative CT scan is made to enable anatomical verification and quantification of the electrode’s position within the cochlear duct, and to compare it with the high-resolution pre-op MRI-CT data (experiment 2) and results of post-operative audiograms.

Post-op implantation, three electrophysiological measurements are performed by applying direct pulse stimulation and field-recording through the CI-electrodes. Experiment 3 assesses the amount of *crosstalk* (interference) between the different contacts by measuring the electrode cross-impedances (ECI) along the array, whereas experiment 4 determines the spread-of-excitation (SOE; ‘*spatial (spectral) resolution*’) of each electrode on the associated auditory nerve responses (De Vos et al., 2018; Bolner et al., 2020; Yang et al., 2020). Finally, binaural audiograms are taken post-op in the clinic (experiment 0b).

Experiment 5 aims to provide a measure for the integrity of the auditory nerve by measuring its population response to single-electrode stimulation. Such electrically evoked compound action potentials (eCAPs) are measured by stimulating different electrodes, and with different current strengths, to determine nerve-activation thresholds and their intensity growth function. Note that an appreciable eCAP only shows up when the excited nerve fibers fire with sufficient synchronicity. Yet, a perceptual response may occur well below the eCAP threshold and may not require strict synchronicity. Thus, although the eCAP test will not be conclusive for assessing neural health, its total absence will be bad news.

Together, these pre- and post-op measures will provide valuable objective prior inputs for the ML algorithms as potential explanatory co-factors for future CI-success and performance variability (Figure 2; see Section 3.5). They can also provide valuable information regarding the effectiveness of identified electrodes and whether certain electrode combinations might better be avoided because of potentially ambiguous interference. If needed, experiments 3–5 and the audiograms can be repeated at a later stage in the lab to verify the electrophysiological stability of AN stimulation.

### 3.2. Reaction times as an objective probe for spectral-temporal sensitivity

In psychophysics, the reaction time of an overt motor response to the detection of a stimulus event has been recognized as a highly informative measure of sensorimotor processing in the brain (Carpenter and Williams, 1995). Reaction-time analyses have been widely used in psychology, in electrophysiological studies, and in visual evoked motor behaviors like eye-, eye-head, or eye-hand coordination responses and have yielded valuable insights into the underlying cortical and sub-cortical neural mechanisms (Carpenter and Williams, 1995; Hanes and Schall, 1996; Gold and Shadlen, 2000; Glimcher, 2003).

Despite the many successful applications and insights, however, reaction times are rarely used in the auditory research field, let alone as a clinical tool for evaluating auditory performance of the hearing impaired. In this paper, we will make the case for introducing the reaction-time paradigm as a valuable model-based tool for auditory clinical evaluation.

**Figure 3:**
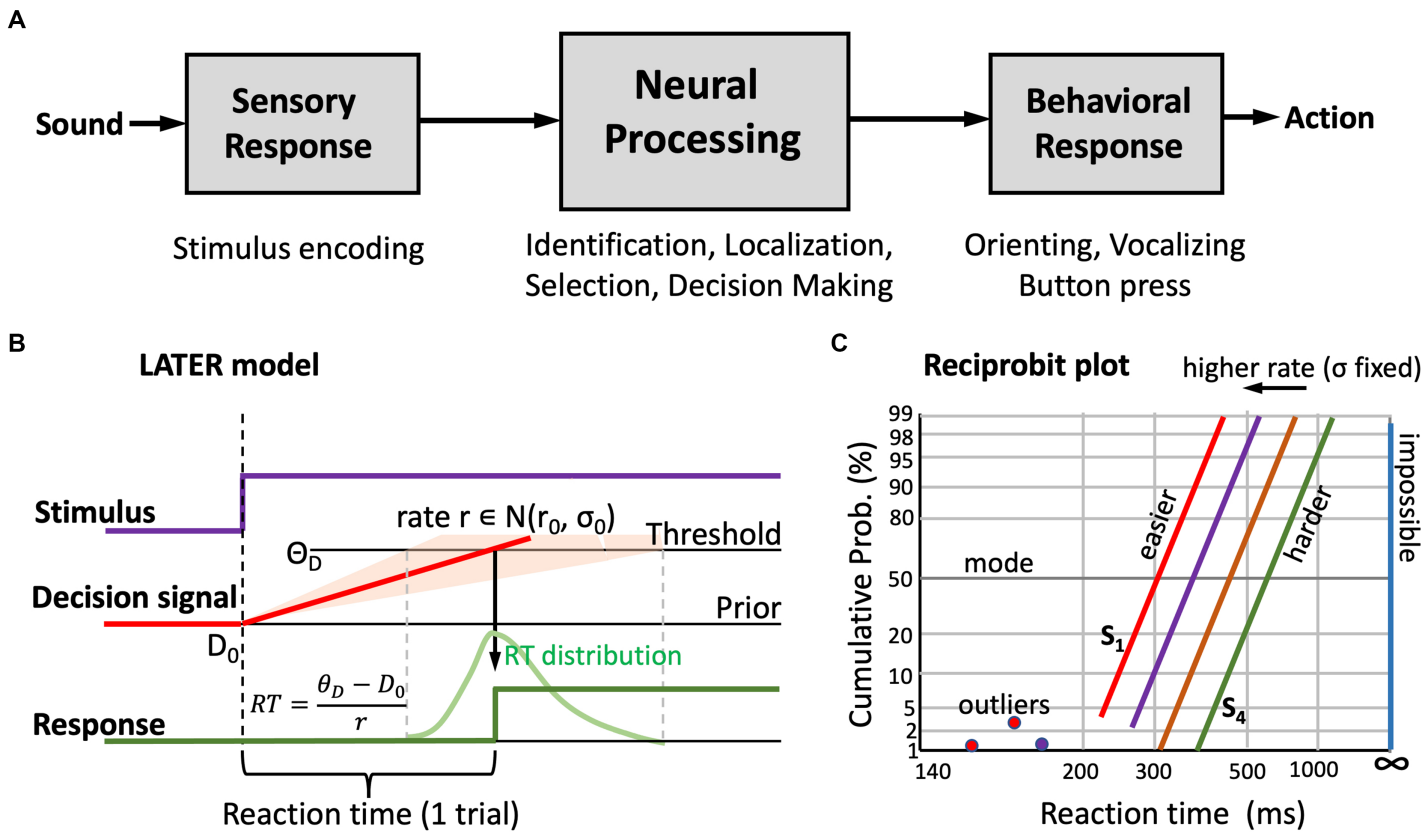
**(A)** General concept of the serial sensorimotor chain: the sensory and motor processing stages have relatively fixed processing times, but central neural processing is highly stochastic. As a result, behavioral responses to a stimulus have a wide distribution of reaction times. **(B)** The “LATER” model (Noorani and Carpenter, 2016) explains the skewed reaction-time distributions by the rise of a stochastic neural signal towards a threshold, in which the rate signals the strength of the sensory evidence. This rate is drawn from a Gaussian distribution (mean r_0_, standard deviation, σ_0_), the width of which reflects internal noise and uncertainty about the stimulus. Based on prior information, or familiarity, the starting level of the signal, D_0_, can be higher or lower; similarly, the threshold level, Θ_D_, is task-dependent (e.g., speed vs. accuracy vs. precision requirements, risk mitigation, etc.). The parameters of the model, and changes therein, systematically affect the reaction-times. **(C)** In a reciprobit plot (inverse reaction time vs. cumulative response probability), a Gaussian distribution becomes a straight line. The slope relates directly to σ_0_, the mode (at 50%) to r_0_. Increasing the mean decision rate leads to parallel leftward shifts of the reciprobit lines. A poor stimulus percept shows as a line that is shifted to the right, with a shallower slope (i.e., increased σ_0_). Outliers (due to inattentiveness, or to prediction) can be readily identified (Noorani and Carpenter, 2016).

Figure 3A illustrates the general concept of the serial sensorimotor response chain (Glimcher, 2003). Stimulus presentation induces a response in sensory receptors and in subsequent sensory neural processing stages, leading up to primary cortical areas. In vision, this ascending sensory pathway takes approximately 60–70 ms of processing time, which is only mildly modulated by factors like stimulus intensity or contrast. For audition, the sensory processing times are markedly shorter: about 20–30 ms pass until the first neural response in primary auditory cortex is observed (Glimcher, 2003; Massoudi et al., 2014).

Also, the motor output stage requires a nearly fixed amount of processing time. For example, the delay between frontal-eye-field (frontal cortex) or superior colliculus (midbrain) activity to the onset of the saccadic eye movement is approximately 15–20 ms. Taken together, the sensory and motor stages for a visual-evoked eye movement use between 75 and 90 ms of neural processing time (Glimcher, 2003), while for an auditory evoked saccade this could be as short as 35–50 ms (Massoudi et al., 2014).

The average reaction time of a saccadic eye movement is close to 200 ms (vision; Carpenter and Williams, 1995) or 170 ms (audition; Hofman et al., 1998; Hofman and Van Opstal, 1998), with a standard deviation close to 50 ms, which is considerably longer and more variable than predicted from the pure sensory and motor delays. Most of the reaction time and its variability is due to processes in the (cortical) central nervous system, where stimulus identification, selection among multiple options, spatial localization, attention, cognitive weighting, and the decision to make the response, together take considerable processing time. In auditory performance, exactly these central processes underly our ability to identify a speaker or a word and understand a selected speech signal in a noisy environment.

Clearly, with a CI or a hearing aid, the sensory input is severely degraded, causing daunting challenges to these central processing stages. Adequate device fitting aims to minimize these challenges. As will be illustrated below, an appropriate assessment of a listener’s reaction times to well-chosen auditory stimuli can provide detailed information about the spectral-temporal sensitivity of the user’s auditory system and provides direct access to the sensory (CI)-to-central mapping in the user’s brain. We will first highlight the underlying theoretical framework and provide some examples from auditory-evoked behaviors to illustrate its potential usefulness for auditory science and clinical use.

#### 3.2.1. The LATER model explains reaction-time distributions

Figure 3B illustrates the central idea of the LATER (*Linear Approach to Threshold with Ergodic Rate*) model, originally proposed by Carpenter and Williams (1995) and Noorani and Carpenter (2016). It explains the skewed reaction-time distributions of visually triggered saccades as the result of a stochastic neural process.

The central assumption of the LATER model is that the variability in reaction times is due to stochastic (Gaussian) noise in the neural rate of accumulating evidence [ $r\in N(r_{0},\sigma_{0}$ )] that leads to the decision threshold, *Θ_D_*, to respond. Importantly, the rate does not start at zero, but at some initial value, *D_0_*, which represents the subject’s prior setting. This initial value can change, depending on stimulus conditions, familiarity, experience, or prior knowledge of the subject. Likewise, the threshold may depend on task requirements, like accuracy, speed, or precision. The assumption is that within a given experimental condition, *D*_0_ and *Θ_D_* remain constant. However, because these levels vary from subject to subject, they account for the idiosyncratic variability of absolute reaction times, and they can be manipulated by changing the experimental conditions (e.g., by adding a level of predictability).

Since the reaction time follows from dividing the distance between threshold and starting level by the selected processing rate, the LATER model predicts that the inverse reaction time, or *promptness*, *p* = 1/RT (s^−1^), has a Gaussian distribution. The green curve in Figure 3B shows the typical skewed distribution of reaction times when plotted on linear time scale. Figure 3C shows the cumulative probability of responses with a given reaction time on so-called *reciprobit* scaling. In this format, the abscissa represents the promptness, shown inverted as actual reaction times (short RT, i.e., high promptness, on the left; long RT, i.e., low promptness, on the right, with infinite RT (i.e., no response, *p* = 0) at the far-right). The ordinate shows the cumulative probability on probit scale.

In reciprobit plots, a Gaussian distribution follows a straight line, where the modus of the reaction times is found at 50% probability, and the slope of the line directly relates to the uncertainty (noise) in the system. Clearly, changes in each of the model parameters lead to specific changes in position and slope of the reciprobit lines. As an example, Figure 3C illustrates the effect of changing the mean, r_0_, of the processing rate in the model, while keeping the internal noise, σ_0_, fixed. This manipulation leads to horizontal shifts of the lines.

**Figure 4:**
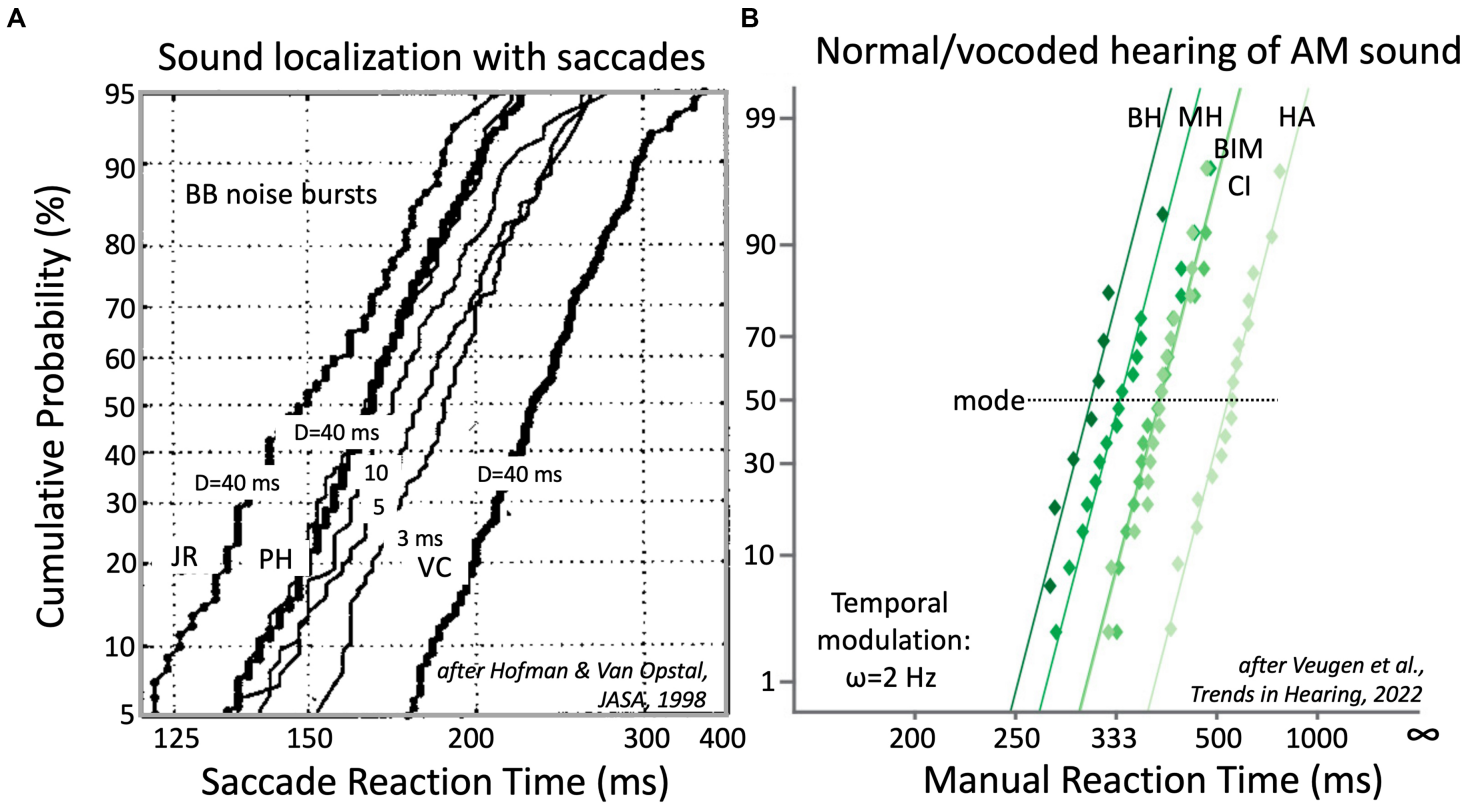
**(A)** Reaction times of saccadic eye movements to broad-band noise bursts, presented in the two-dimensional frontal hemifield, follow near-straight reciprobit lines that vary systematically with sound duration, D. Bold lines: data for D = 40 ms bursts (at 65 dB SPL) for three NH subjects. Note the idiosyncratic differences. For PH, results are also shown for 20, 10, 5 and 3 ms bursts (thin lines). Note that the lines are approximately parallel and that they shift rightwards with decreasing D (Hofman et al., 1998). **(B)** Button-press reaction times to a 2 Hz amplitude modulation of a broadband sound depend on the quality of hearing. This is shown for NH listeners subjected to normal binaural hearing (BH), normal monaural hearing (MH), and three vocoded hearing conditions: bimodal (BIM = CI + HA), unilateral CI (1–8 kHz, in 6 bands) and unilateral HA (0.25–1.5 kHz). Adapted from Veugen et al. (2022).

*Auditory reaction times.* As an example, Figure 4 demonstrates that also the reaction times of auditory-evoked motor responses follow these strict relationships and that even subtle manipulations of the acoustic input, like reducing the duration of a broadband sound burst from 5 ms to 3 ms (Hofman et al., 1998; Figure 4A) or listening to a modulated sound with either two ears vs. monaurally (Figure 4B; Veugen et al., 2022), systematically and reliably affect the reciprobit lines.

Importantly, application of the LATER framework is independent of the type of motor response or of the specific motor-response task. It can be equally well applied to saccadic eye movements in a sound-localization test (Figure 4A), as to a manual button press task to the detection of the onset of a temporal modulation, as in Figure 4B. Below, we describe the different reaction-time tests that are proposed in Table 1, and that enable the characterization of the spectral-temporal sensitivity of the listener.

#### 3.2.2. Advantages of reaction times

Using reaction times to assess the integrity of signal processing in the auditory system has several advantages above other behavioral outcome measures, such as threshold psychometrics, alternative forced-choice methods, or speech-reception thresholds and scores.

- It’s a simple stimulus-*detection* task, which unlike alternative forced-choice methods, does not require memory resources to compare different stimuli presented at different times.
- A rapid response (order of a few hundred milliseconds) that is not contaminated by the contribution of higher-level cognitive factors.
- The task (‘*respond as fast as possible to the stimulus as soon as you perceive its appearance*’) requires little instruction or training: children, and even monkeys (Massoudi et al., 2014; Van der Willigen et al., 2023) can do the task.
- Reaction times can be measured at suprathreshold stimulus levels. Task execution requires little attentional effort of the participant and thus prevents rapid mental fatigue.
- In contrast to threshold measurements, trial execution is fast (order of a few seconds), thus allowing for the collection of many (hundreds) trials in a recording session for reliable statistics.
- In contrast to threshold measurements, trials for different stimuli can be interspersed, preventing adaptation and habituation effects or predictive factors.
- Results are reproducible and remain stable over time. Data can be collected and pooled over multiple sessions.
- Reaction times provide a sensitive measure for acoustic processing: even minor acoustic manipulations lead to measurable and systematic shifts in the reciprobit plots (e.g., Figure 4). We have also observed this for direct electrode-stimulation in CI users.
- Outliers (but also experimental artefacts) can be readily identified as deviations from the straight lines.
- Because of the strict distributions of stimulus-evoked responses, one can be practically sure that whenever the response is on or near the straight reciprobit line, the subject indeed perceived the stimulus, and reacted in response to the stimulus proper.
- By randomizing stimulus presentations, it is straightforward to prevent nonstationary behaviors like perceptual learning, prediction, or adaptation during the experiments.

Finally, reaction-time distributions have a solid theoretical underpinning, and they provide valuable information about the underlying sensorimotor system (Figure 3). For example, possible involvement of multiple sub-systems (like bottom-up vs. top-down mechanisms; Carpenter and Williams, 1995), different processing pathways (e.g., frequency channels, ILD, ITD, temporal, spectral, monaural vs. binaural, cortical vs. subcortical, etc.), uncertainty about the stimulus (the width of the distribution), but also listening effort (cognitive load) can be assessed with reaction time analyses (Carpenter and Williams, 1995; Hofman et al., 1998; Veugen, 2017; Veugen et al., 2022). Indeed, the latter could serve as a good alternative for the highly noisy and slow pupil-dilation method that is currently measured with video eye-tracker techniques (Zekfeld et al., 2018).

Table 1 proposes three different experiments to measure the reaction times of CI recipients. First, in response to acoustic presentation of moving spectrotemporal ripples (experiment 5), and second, in response to changes in the patterns of direct electrical pulse stimulation on the electrodes of the implant (experiments 11 and 12). We first briefly introduce the moving spectrotemporal ripple.

#### 3.2.3. The moving spectrotemporal ripple

**Figure 5:**
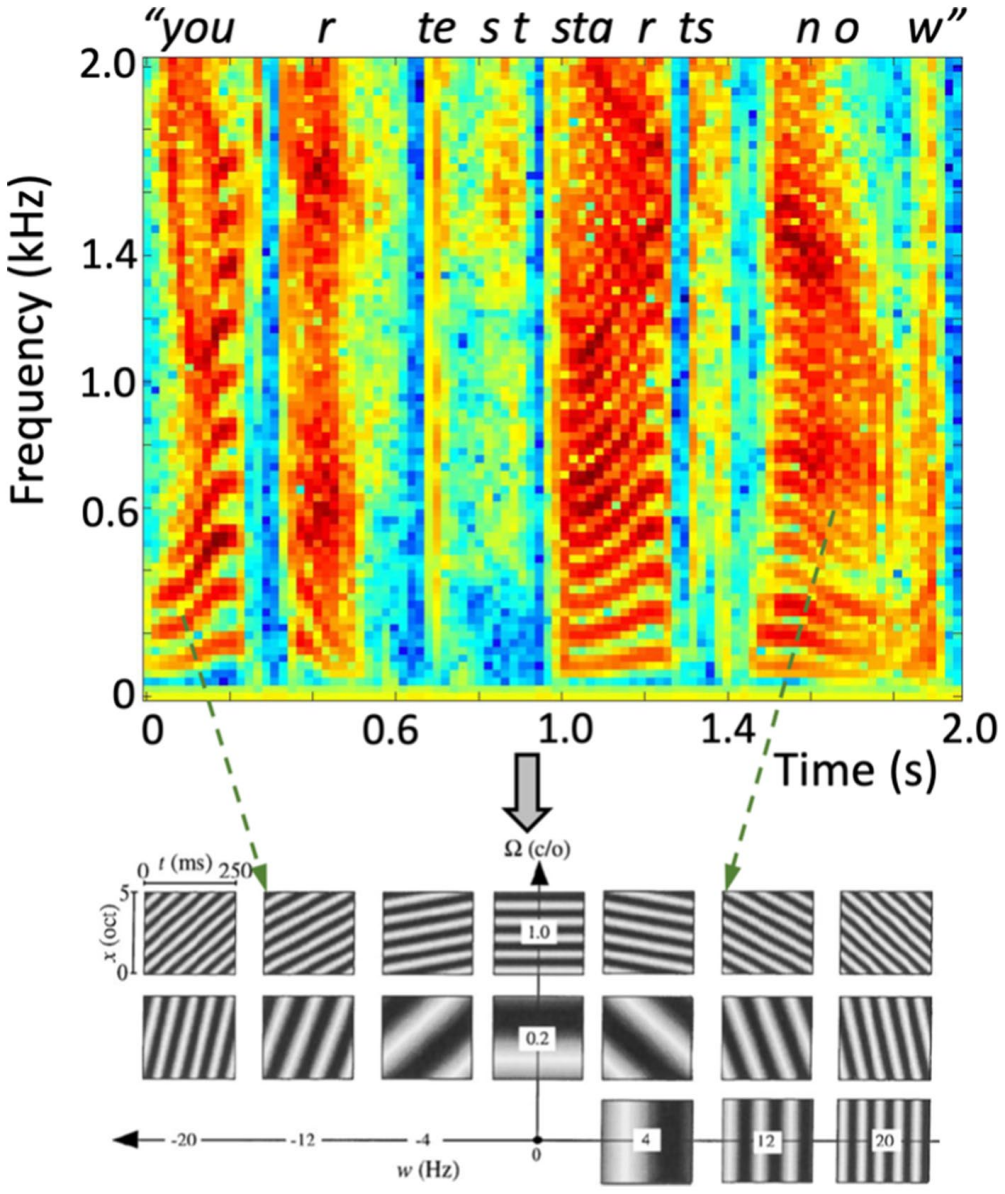
(Top) Spectrotemporal representation (sonogram) of the amplitude of a human speech utterance “Your Test Starts Now”, containing a complex dynamic sequence of rising and lowering harmonic complexes (the vowels, e.g., at 0–0.25 s, 1.0–1.2 s, 1.45–1.95 s), interspersed with broad-band nonharmonic fricatives (e.g., at 0.7, 1.35 s). (Bottom) Dynamic ST ripples can represent the full space of joint spectrotemporal modulations at any time-frequency interval. Here, 17 examples of 0.25 s with different densities and velocities are shown over the full bandwidth of 5 octaves. Two instances in the recorded speech signal point at roughly corresponding ripples (dashed arrows).

**Figure 6:**
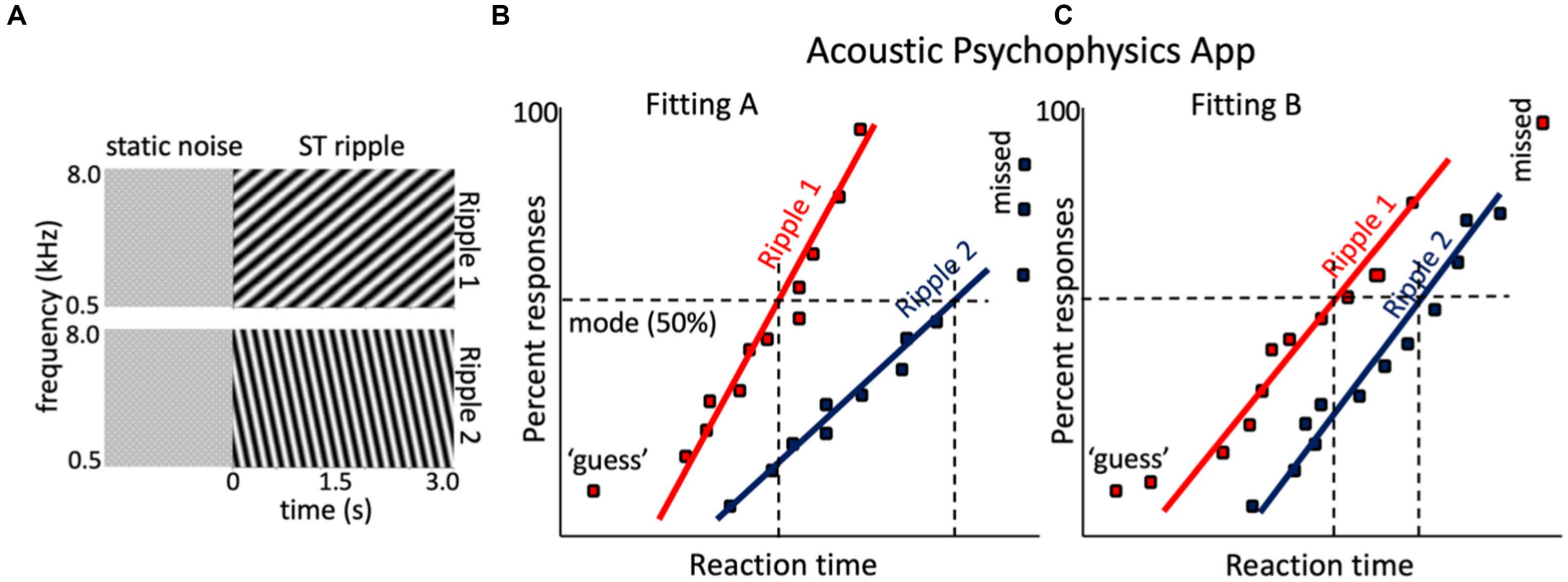
Hypothetical result for the spectrotemporal ripple reaction-time experiment of a given CI user with two different CI fittings. **(A)** Two sonograms of example broadband dynamic spectrotemporal ripples: ripple 1 is a downward ST modulation (ω, Ω) = (4 Hz, −1.2 c/o), and ripple 2 is upward (ω,Ω) = (8 Hz, +0.6 c/o). Both are preceded by a (random) period of static noise. **(B)** Hypothetical reciprobit results for the two ripples of the listener, with the CI in the Fitting A mode. Ripple 1 is easier to perceive then ripple 2, which yields a long-latency mode and shallow slope. Sometimes the listener even failed to respond to ripple 2 (‘missed’). **(C)** After changing the processor to Fitting B, the responses to the two ripples changed. Now ripple 1 is detected later with Fitting A and with more variability, but performance for ripple 2 has improved.

A moving spectrotemporal (ST) ripple is described by the envelope modulation of a broadband or band-limited carrier of duration *D_RIP_* seconds and bandwidth, *Δf = [f_min_, f_max_]*, which consists of either Gaussian white noise, pink noise, or a superposition of finely space tones with randomize phases (e.g., at 1/20 octave intervals; Chi et al., 1999; Elliott and Theunissen, 2009; Versnel et al., 2009; Veugen, 2017; Van der Willigen et al., 2023). The ripple’s ST modulation is parameterized by:


(1) R(ω,Ω)=1+ΔM.cos(2π(Ω.x±ω.t))

with *x* the spectral position of the ripple in octaves above the lowest frequency in the stimulus (e.g., when f_min_ = 500 Hz, *x* = 1 corresponds to *f* = 1.0 kHz) and *t* is time in seconds since the ripple onset. ΔM ∊ [0, 1] is the modulation depth (typically taken >0.6, which is suprathreshold), Ω (in cycles/octave) is the spectral *density* of the ripple, and ω (in Hz) is the ripple’s temporal *velocity*. For ω/Ω < 0 the ripple’s movement direction is an upward spectral sweep, otherwise it’s a downward spectral modulation (Figure 5, bottom). Figure 6A shows two examples of moving ST ripples: ripple 1 is an upward-moving ripple with [ω, Ω] = [4 Hz, −1.2 c/o], and ripple 2 moves downward with [ω, Ω] = [8 Hz, +0.6 c/o].

As illustrated in Figure 5, moving ST ripples can be considered as the elementary building blocks of most natural sounds, including speech: any speech signal can be decomposed into a unique dynamic spectral- and temporal summation of short band-limited and broadband ST snippets, in the same way that sines and cosines underlie Fourier decomposition and the sonogram. As such, human speech can be considered as a dynamic sum of such ripples over spectrum and time, typically confined to temporal modulations (velocities) |ω|≲ 7–10 Hz, and spectral modulations (densities)|Ω|≲ 1.0 cycles/octave.

While speech-perception tests unavoidably address the involvement of higher cognitive functions (language skills, prediction, memory, familiarity, lexicon, etc.), on top of the bottom-up spectrotemporal analysis from the ascending auditory pathways, the advantage of ST ripples is that they have no cognitive meaning, yet jointly encompass the full spectrotemporal dynamics of natural speech. Thus, measuring responses to ST ripples mainly probes the integrity of the ascending spectrotemporal processing pathways, without cognitive interference from higher neural mechanisms.

**The ST-ripple RT test.** In a reaction-time test with ST-ripples, the ripple is preceded by a period of flat noise of the same carrier and mean sound level as the ripple for a randomized duration, *D_NOISE_* (e.g., between [1.0–3.0 s]; Versnel et al., 2009; Massoudi et al., 2014; Veugen, 2017; Veugen et al., 2022; Van der Willigen et al., 2023). The listener must press a button (or touch a keyboard’s space bar, handle, or smartphone screen) as soon as the ripple onset is detected. By combining the RT data across different ST-ripples, all randomly interleaved in an experimental session, the ST sensitivity and resolution of the listener’s auditory perceptual system can eventually be determined (see also below).

In case of a CI recipient, ST sensitivity is recorded for each applied fitting. The underlying idea is that changing specific parameters in the CI fitting will affect the acoustic processing of the implant and will thus lead to specific changes in the RT distributions (reciprobit lines) for certain ripples, such as illustrated in Figure 4B. In our own experiments, we have so far noted that, typically about 20 trials per ripple suffice to obtain a reliable estimate of the reciprobit line, also for CI users (Veugen, 2017; Veugen et al., 2022).

The rationale of the ST ripple test is tentatively illustrated in Figures 6B,C. The two ripples in Figure 6A were presented to a hypothetical CI user tested with two different fittings, Fitting A, vs. Fitting B. With Fitting A, ripple 1 is detected well, but the listener has problems with ripple 2 (often misses its detection altogether). With Fitting B, ripple 2 is perceived much better and more reliably [slope increased, indicating less variability (uncertainty)], and the mode shifted leftward, indicating easier detection. Simply put, if ripple 2 would contain essential spectrotemporal modulations found in human speech (Elliott and Theunissen, 2009), it is expected that Fitting B yields better speech comprehension than Fitting A (Chi et al., 1999; Veugen, 2017).

#### 3.2.4. The ST modulation transfer function

**Figure 7:**
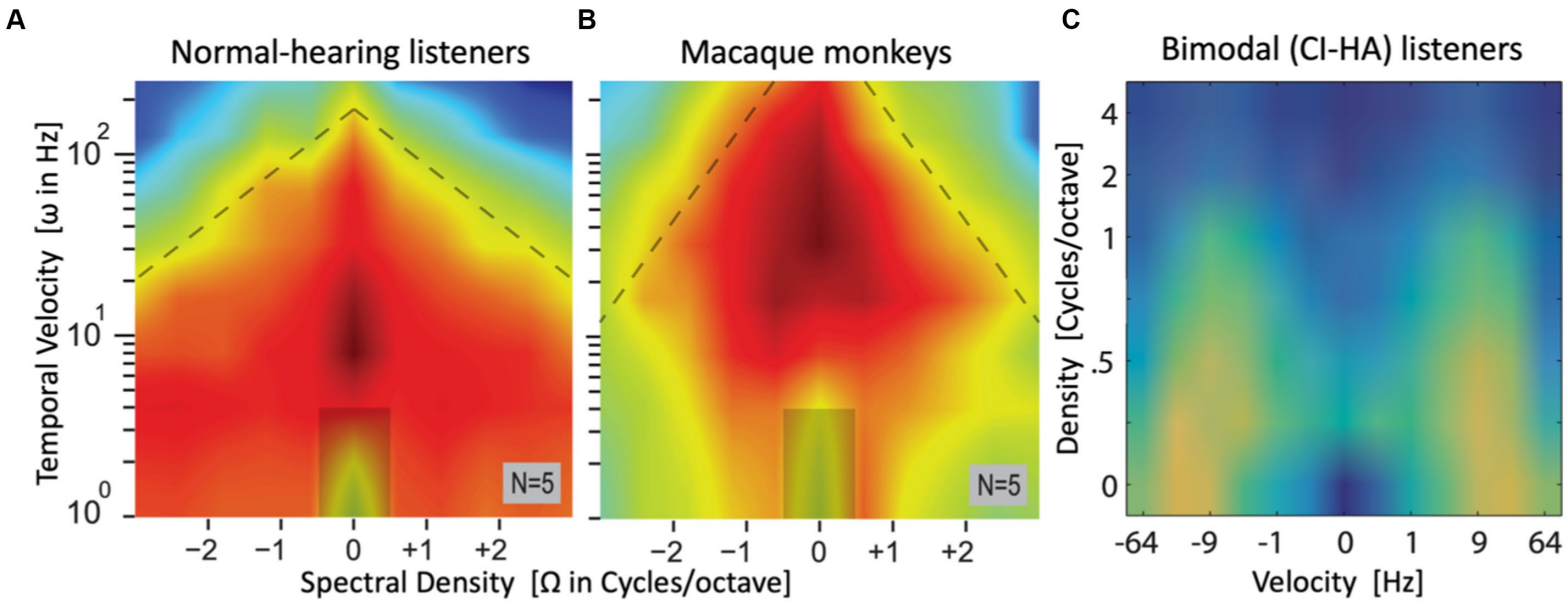
Sneak preview of complete (averaged) spectral-temporal modulation transfer functions, obtained from the modes of the manual promptness distributions for a large set of upward and downward spectral-temporal ripples, spanning spectral densities between [−3, +3] cycles/octave and velocities from 0–256 Hz in normal-hearing listeners **(A)** and macaque monkeys **(B)** [both adapted from Van der Willigen et al. (2023)], and [−64, +64] Hz, 0–4 c/o in bimodal listeners **(C)** [adapted from Veugen (2017)]. Note that abscissa and ordinate of the patient data are flipped. Hot colors (red/yellow) indicate high sensitivity (short RTs, high promptness), green/blue is low sensitivity (long RTs, low promptness). The ripple at [0,0] is a catch stimulus without spectral-temporal modulation. In all three cases, highest sensitivity is found around Ω = 0 c/o (NH humans and patients: ω ~ 7–10 Hz; monkeys: ω ~ 20–50 Hz).

Figure 7 provides a sneak preview of a quantitative assessment of the spectrotemporal sensitivity of the auditory-perceptual system by analyzing the results from the reaction-time ST-ripple paradigm to many ripples, covering a wide range in the ripple velocity/density plane. It shows the average spectrotemporal modulation transfer functions, MTF_ST_[Ω, ω], for three different types of listeners: five normal-hearing (NH) adult human listeners (Figure 7A), five rhesus monkeys, trained to perform in the same reaction-time paradigms as the NH humans (Figure 7B), and eleven bimodal CI-HA listeners (Figure 5C). The panels show (in color code) the mode of the promptness distributions in the [Ω, ω] plane (Figures 7A,B) or [ω, Ω] plane (Figure 7C) as measured for about 90 upward (negative abscissa) and downward (positive) ripples, all presented at a suprathreshold modulation depth of ΔM = 0.8.

Results such as these contain a wealth of information, which may be extracted by subsequent analyses. For example, by making two cross-sections through these plots one can estimate the listeners’ temporal MTF (at Ω = 0), and spectral MTF (at ω = 0). Furthermore, one can assess potential inter-dependencies between the spectral and temporal processing streams in the auditory system (their *(in)separability*), or different sensitivities for upward vs. downward spectral modulations, by analyzing the properties of the full MTF_ST_ matrix. Also, the *slopes* of the reciprobit lines convey valuable information about ST sensitivity of the listener, which is not included in these MTF plots. As these additional analyses are beyond the scope of this paper, the interested reader is referred to (Chi et al., 1999; Elliott and Theunissen, 2009; Veugen et al., 2022).

Note that obtaining MTFs such as these will require several experimental sessions. For example, the data in Figures 7A,B comprised 88 different ripples (including the catch stimulus at [0,0]): 8 temporal modulations (0-4-8 …·128–256 Hz) × 11 spectral modulations (between −3.0 and +3.0 cycles/octave in 0.6 c/o steps). This would amount to about 1760 trials (20 per ripple) for each fitting (~3 h net measuring time). Fortunately, this many responses can be measured across different sessions, spread over several days, or even weeks. This becomes a feasible exercise when the data can be obtained in the user’s own home environment.

### 3.3. Direct electrode stimulation

With dedicated software provided by the hearing-aid manufacturer (e.g., Advanced Bionics’ program “*BEDCS*”), custom-defined stimulation pulse patterns can be delivered directly to single or combined sets of electrodes to bypass the CI’s speech processor and fitting.

**Figure 8:**
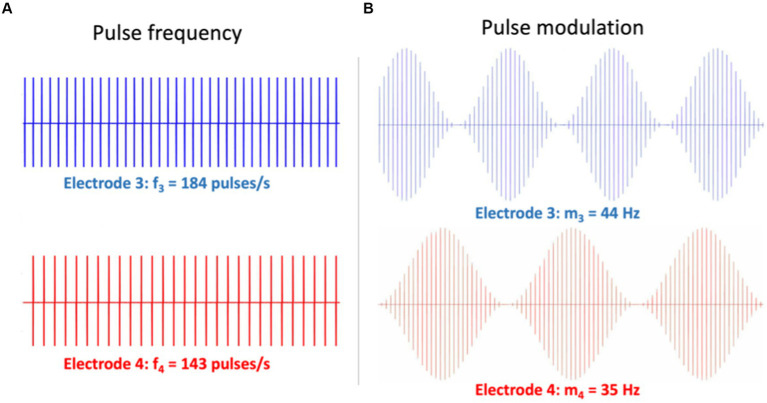
Illustration of two different electrode stimulation patterns for Exp. 11 in Table 1. Simultaneous stimulation is provided to two electrodes (e.g., nrs. 3 and 4). **(A)** Different pulse rates with a constant pulse amplitude. The 2nd-order eASSR for these patterns is expected at Δf = 41 Hz. **(B)** The same pulse-rate carrier, but different modulation frequencies on the pulse amplitudes. Here, the 2nd-order eASSR is expected at Δfm = 9 Hz. Note that in Exp. 14, the electrodes may refer to the same stimulation channel in different ears (e.g., Electrode 3 left and 3 right).

These experiments should be performed in the clinic, or in a dedicated laboratory. Figure 8 shows two examples for experiments 11 and 14 (Table 1). In these experiments, the participant remains passive and does not make a perceptual judgment or a motor response, as the goal is to measure potential nonlinear distortion products, which can be observed in the frequency spectrum of the cortical EEG.

The spectral distortion components form the so-called *Auditory Steady-State Response* [ASSR, when evoked acoustically (Picton et al., 2003; Schwarz and Taylor, 2005; Picton, 2011; Wang et al., 2021; Noordanus et al., in prep.); or eASSR when evoked electrically (Hoppe et al., 2018)]. The pattern of (e)ASSR spectral components is due to nonlinear neural interactions in the auditory system that are evoked by the CI’s pulse patterns. As such, the presence of an eASSR is a clear signature of neural processing in the CI-user’s auditory system.

#### 3.3.1. Spectral analysis of nonlinear distortions in the EEG

Clinically, the ASSR is used to assess auditory function and potential hearing loss in newborns and children, who are unable to give a reliable perceptual response (Luts et al., 2006). Importantly, a significant ASSR at the modulation frequency can be directly related to the carrier frequency of the auditory stimulus, and its strength correlates to hearing thresholds at the carrier (Picton, 2011). By using different carrier frequencies, one can simultaneously determine hearing thresholds at different carriers (Luts et al., 2006), making the ASSR an interesting objective measure for auditory processing. The ASSR can also been used to study *binaural integration* with binaural beat stimuli [one tone in the left ear and another tone with a nearby frequency in the right ear (Schwarz and Taylor, 2005)].

The ASSR reflects *nonlinear* processing within the auditory system, as nonlinearities create frequency distortion products that are not part of the input spectrum. The theoretically possible nonlinear distortion products between M harmonic carriers, (f_1_, f_2_, ⋯, f_M_), are:


(2) $$f_{NL}=\;|\left(\pm n_{1}.f_{1}\pm n_{2}.f_{2}\pm \cdots \pm n_{M}.f_{M}\;\right)|\;\mathrm{with}\;\;n_{i}\in \left\{0,\mathbb{N}\right\}$$

where the *order* of the nonlinear component is given by $\sum_{M}^{k=1}\left|n_{k}\right|$ . Note that the total number of distortion components is vast. E.g., it can be shown that with only three carrier frequencies, 83 distortion products are generated up to the 4th order (Veugen et al., 2016)! As an example, *∆f_NL_ = |3f_3_ ± f_1_|* and *∆f_NL_ = |2f_3_ ± 2f_2_|* are due to 4^th^-order nonlinear interactions.

A second-order monaural distortion in the ASSR, observed as a difference frequency between two carriers, *∆f_NL_ = |f_2_ - f_1_*|, is already introduced in the cochlea by nonlinear compression through outer hair cells, combined with inner hair cell rectification and synaptic transduction to the auditory nerve (Picton et al., 2003; Van der Heijden and Joris, 2003; Picton, 2011). In normal hearing, distortion components around 40 Hz are especially prominent (Picton et al., 2003; Picton, 2011).

Clearly, the cochlea-to-nerve interactions in normal hearing are severely compromised in the CI user, where inner- and outer-hair cell functions are no longer present. It is therefore of interest to observe similar distortion products in the EEG in response to acoustic stimulation with a particular fitting (experiment 15) or elicited by stimulating different electrode pulse trains that mimic the situation of tones presented at nearby frequencies (experiment 11).

**Figure 9:**
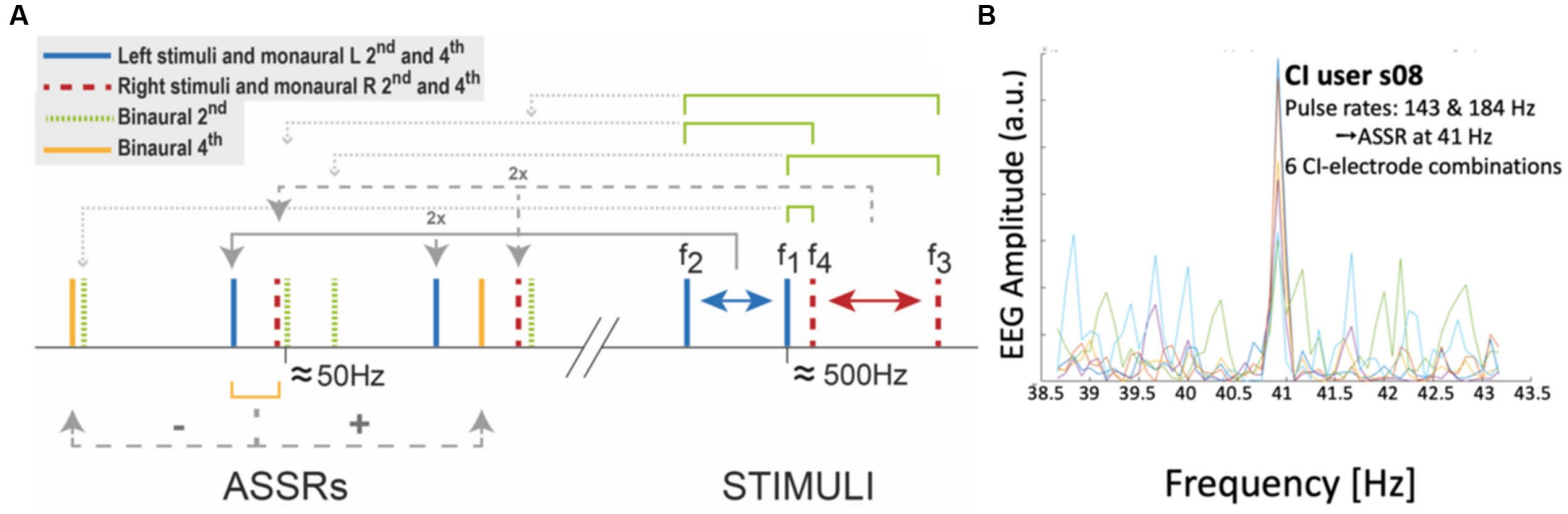
Auditory steady-state responses (ASSRs) in the EEG reflects nonlinear processing in the auditory system. **(A)** Selection of monaural and binaural nonlinear distortion products generated by two pairs of tones presented to the left (461 and 500 Hz) and right (504 and 537 Hz) ear, respectively. **(B)** Proof of principle of measuring the ASSR in a CI user (Exp. 11 in Table 1). The response shows a peak at the 41 Hz difference frequency in the EEG of CI-user s08, generated by two different direct-stimulation pulse rates of 143 and 184 Hz, respectively (see Figure 8A), presented at six different electrode pairs (each colored trace corresponds to a different pair). Unpublished data.

Figure 9A highlights nine of these low-frequency distortions (<100 Hz), expected to be generated by two pairs of incommensurable carriers around 500 Hz, and presented to either ear [note that many more components below 100 Hz and up to the 8th-order can be predicted for these frequencies (Wang et al., 2021; Noordanus et al., in prep.)]. To detect the spectral components in the EEG of an ASSR experiment, the Fourier spectrum should be analyzed at sub-Hz spectral resolution, typically up to about 120 Hz, which is within the bandwidth of regular EEG amplifiers (Palmer and Summerfield, 2002; Picton et al., 2003; Wang et al., 2021; Noordanus et al., in prep.). Figure 9B shows a strong eASSR at 41 Hz in a CI user to the pulse trains of Figure 8A, which could be measured without the need for stimulus artefact removal (Hofmann and Wouters, 2012; Carlyon et al., 2021).

Note that nonlinear *binaural* responses (like the green and yellow components in Figure 9A) can only be created *upstream* in the central auditory system. It would therefore be of great interest to demonstrate binaural ASSRs in *bilateral CI users*, as these would provide unequivocal objective evidence for true binaural central integration. This possibility is explored in experiments 14 and 15.

#### 3.3.2. The auditory change complex in the EEG evoked potential

One can reliably record cortical evoked potentials on scalp EEG electrodes, also in CI recipients (Van Heteren et al., 2022; Vonck et al., 2022). The auditory change complex (ACC) is the averaged EEG response (typically over 50–100 stimulus repetitions) to a sudden change in the sound, like the change in frequency of a pure tone. Recent studies have indicated that the latency of the evoked P1-N1-P2 complex in evoked potentials to low-frequency tones correlates well (*r^2^* ~ 0.7, or higher) with speech-reception thresholds in noise and with frequency-discrimination thresholds (Van Heteren et al., 2022; Vonck et al., 2022). It may be expected that changes in the fitting will affect the ACC patterns and could thus provide valuable objective outcome information on expected SRTs. Experiment 16 will systematically exploit this possibility by quantifying the ACC’s for different frequency changes (Δf) from different base frequencies (f_0_). The base frequencies will be selected to correspond to the first 5 apical electrodes. As the mean N1-latency of the ACC correlates with SRT in noise, the goal to predict how a change in fitting affects the mean ACC N1-latency.

#### 3.3.3. Electrical evoked reaction-time psychophysics

In experiments 12 and 13, the direct electrode stimulation paradigm is used to evoke a rapid manual response from the CI-user. In experiment 12, a constant-frequency and amplitude pulse train (e.g., 180 Hz) on a given electrode unexpectedly changes to a different frequency (e.g., 220 Hz). The participant is asked to react as fast as possible to the perceived change. This experiment addresses pulse-pattern sensitivity within a single frequency channel.

In experiment 13, two nearby electrodes are stimulated with the same frequency pulse-trains, after which one of the electrodes changes unexpectedly to a different one (e.g., stimulation of electrodes 4 + 5 changes to stimulating 5 + 6; i.e., a ‘jump’ from electrode 4 to electrode 6 in the presence of a background, produced by 5). Again, the CI-user responds with a button press as soon as the change in the pulse patterns (possibly reminiscent to a change in perceived ‘pitch’) is heard. Note that this RT-eACC experiment is the electrical equivalent of the ACC recording paradigm (experiment 16) and the frequency discrimination psychometrics of experiment 8 (Van Heteren et al., 2022; Vonck et al., 2022).

**Figure 10:**
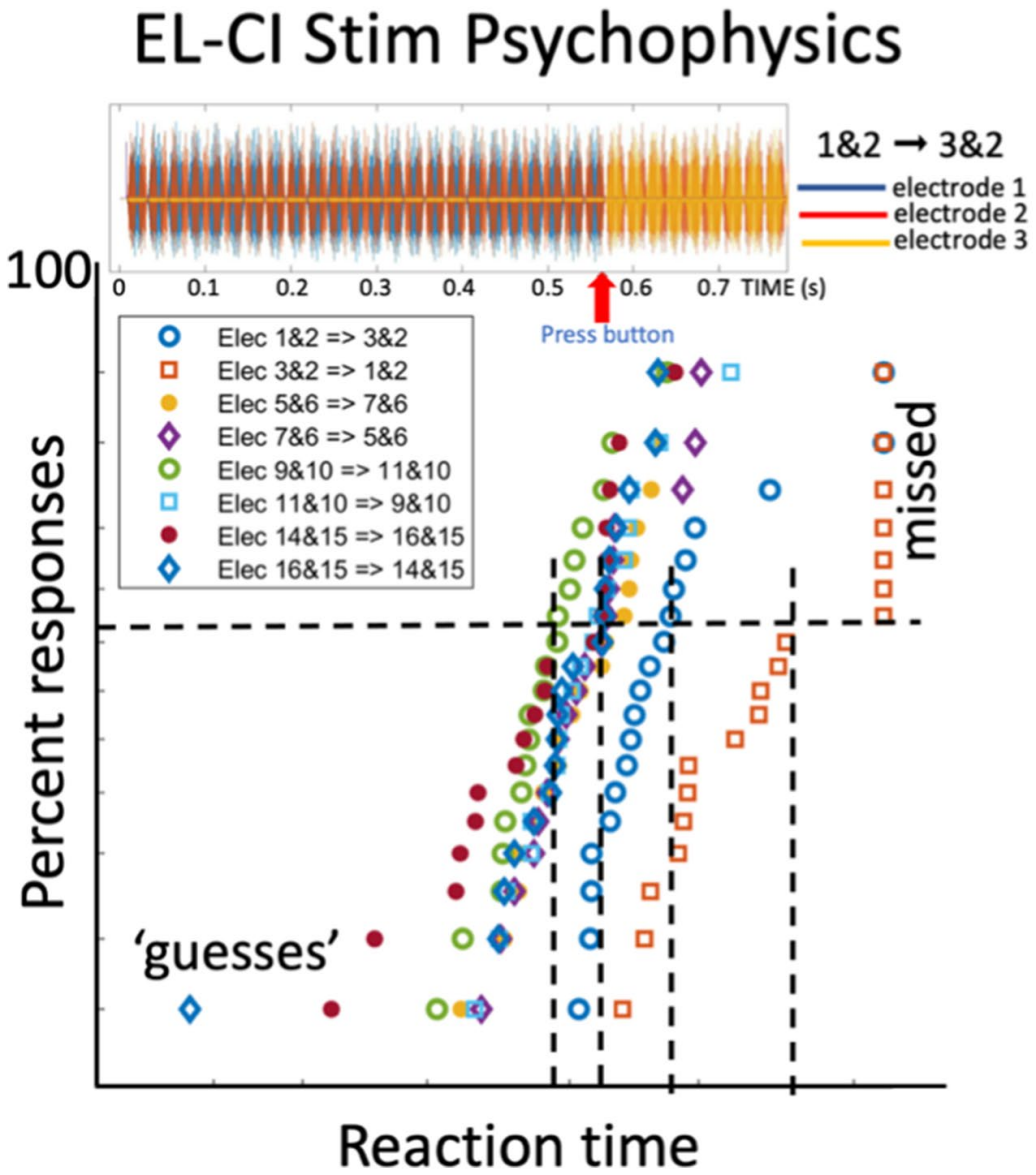
Reaction times of a CI user in response to a perceived change in the electrode stimulation pattern (Exp. 11 in Table 1). Top plot shows the stimulus pulses delivered to electrode 1 and 2 (for 550 ms) followed by electrodes 3 and 2 at t = 550 ms. The listener responds as soon as possible with a button press to the perceived change. The bottom figure shows the reciprobit data for eight different electrode-change combinations. The blue open dots correspond to the stimulus shown at the top. Note the parallel straight lines. Unpublished data.

Figure 10 shows reaction-time results of experiment 13 for a CI-user for eight different electrode combinations. The response patterns nicely resemble the reciprobit RT results to acoustically presented ST ripples (*cf.* Figures 4B–7). This indicates that the listener’s auditory system had different processing rates for the varying acoustic percepts associated with the different electrode configurations. Such RT patterns provide novel objective information of the CI-user’s auditory processing streams in different frequency (electrode) channels, while circumventing the CI’s speech processor and its fitting (Figure 2).

### 3.4. Binaural integration

Binaural integration underlies our capacity for spatial hearing, as well as the ability to segregate a sound-source from a noisy background (‘*spatial-release-from-masking*’; Avan et al., 2015; Van Opstal, 2016; Veugen et al., 2016; Rak et al., 2019; Ausili et al., 2020). True binaural integration in normal-hearing listeners exceeds the summed effects of either ear as it involves central neural integration of the signals in binaural auditory nuclei [e.g., brainstem Superior Olive, midbrain Inferior Colliculus (Versnel et al., 2009), the Medial Geniculate Body, and Auditory Cortex (Massoudi et al., 2014; Wang et al., 2021; Noordanus et al., in prep.)].

To enable true binaural integration in bilateral CI users, however, signals should be precisely timed (at sub-ms synchronization), provide valid interaural sound-level differences within a narrow dynamic range, and provide matched frequency-allocation tables (see Introduction). If inappropriate, bilateral summation may even *hamper* hearing. Thus, the fitting problem for a unilateral CI applies to either ear *plus adds a new set of binaural integration requirements*. Experiments 9, 10, 11 and 15 address this problem.

*ILD/ITD sensitivity.* Experiment 8 determines ITD and ILD frequency-specific psychometric sensitivity curves for narrowband sounds. In these experiments, listeners make a discrimination judgment by indicating with a left/right button press (which can also be taken as a reaction-time paradigm by stressing speed, but this is not required) whether a perceived binaural sound (presented to the left and right speech processers over a Bluetooth connection) is perceived to the left or right of their midsagittal plane. The parameters to be changed are the ITD, ILD and IFD (interaural frequency difference).

*Free-field sound localization.* To investigate free-field binaural integration in bilateral recipients, Exp. 10 measures their active sound-localization orienting head movements to brief (<100 ms) sounds presented at different azimuth directions in the horizontal plane with respect to the head (Agterberg et al., 2011, 2012, 2014; Veugen et al., 2016; Ausili et al., 2020) (note that spectral elevation cues (Hofman et al., 1998; Van Opstal, 2016) will remain inaccessible to CI users because of their poor spectral resolution and limited frequency range). It is important to randomly vary the absolute sound levels of the stimuli over a sufficient range (i.e., 20–25 dB) to reliably identify the contribution of the monaural head-shadow effect to their localization estimates (Van Wanrooij and Van Opstal, 2004; Agterberg et al., 2011, 2014). Our experience with patient sound-localization experiments typically has yielded consistent and reproducible (multiple) regression results (Van Wanrooij and Van Opstal, 2004; Agterberg et al., 2011, 2012, 2014; Veugen et al., 2016, 2022; Ausili et al., 2020; Sharma et al., 2023). In the data analysis, the localization response is described as a bi-linear function of the actual stimulus location (*T_AZI_*; true sound-localization sensitivity), and the stimulus level (*L_SND_*; head-shadow sensitivity; Van Wanrooij and Van Opstal, 2004; Agterberg et al., 2012):


(3) $$R_{AZI}=b_{0}+\alpha .T_{AZI}+\lambda .L_{SND}$$

with b_0_ the response bias (in deg), α the azimuth response gain (dimensionless) and λ the level response gain (in deg./dB). An ideal localization response would yield b_0_ = λ = 0, and α = 1. In case the user relies entirely on the head shadow, b_0_ and λ deviate significantly from zero, with α ≈ 0. To assess the relative contributions of ITD vs. ILD cues, the sound sources can be given different spectral bandwidths: low-pass filtered noise (LP: 0.25–1.5 kHz) for ITD sensitivity, high-pass filtered noise (HP: 3.0–10 kHz) for ILD sensitivity, and broadband noise (BB: 0.25–10 kHz), to determine potential interactions between these two binaural processing streams (Agterberg et al., 2011, 2012, 2014; Ausili et al., 2020). The regression of Eq. (3) is performed separately for the different sound types. Typically, about 30–50 localization responses per stimulus type suffice for a reliable regression over an azimuth range of about [−75, +75] deg., which can be acquired within a 15 min recording session (roughly, 5 s/trial).

**Binaural ASSRs**. Finally, experiment 11 investigates the presence of binaural eASSRs in the EEG by presenting the CI-pulse patterns of Figure 9 in similar channels at either ear. Binaural acoustic ASSRs can be determined by paired tonal sound stimulation at either ear in experiment 15 (Wang et al., 2021; Noordanus et al., in prep.). In this case, the effects of the fitting on the occurrence of binaural beats, a clear indicator for true binaural integration, can be quantitatively assessed.

### 3.5. Machine learning

Once the data are collected, a major challenge will be to combine all this knowledge into a coherent model that allows the clinician to predict the perceptual outcomes of an individual CI-user after applying a particular change to the fitting of the CI.

**Figure 11:**
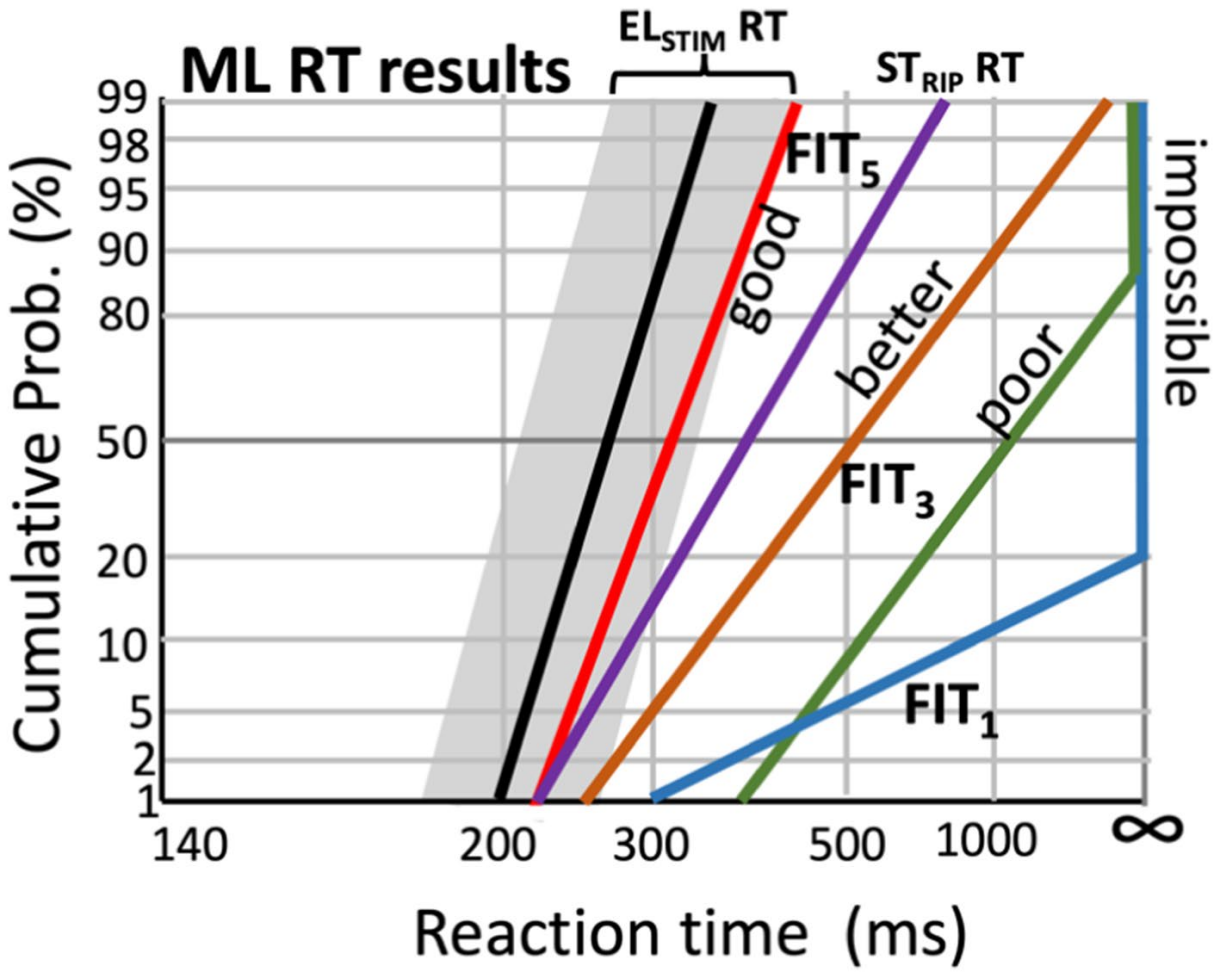
Hypothetical effect of changing the CI fitting parameters on speech-ripple evoked RT distributions (experiment 7), by the machine-learning algorithm that aims to minimize the difference (‘cost’) between the acoustic-evoked reaction times (ST_RIP_ RT) and those elicited by the electrical stimulation (EL_STIM_ RT; Experiments 12 and 13; black line, with the range for the best electrode configurations indicated in gray; see Figure 6).

It is to be expected that the functional relationships between the fitting, the EL-AN interface, and the different modalities of the acquired response data (reaction times, EEG responses, anatomy, ECochG, eCAP, SOE and ECI, and speech performance) will be quite complex, interdependent, and not analytically tractable through simple regression analyses. For that reason, advanced machine-learning (ML) methods will have to be developed to uncover the underlying structure in these rich data sets (Büchner et al., 2015; Meeuws et al., 2017; Crowson et al., 2020; Skidmore et al., 2021). The goal of the ML algorithm will be to provide a fitting advice that leads to better speech-in-noise performance. For example, it has recently been suggested (Van Heteren et al., 2022; Vonck et al., 2022) that the N1-latency of the ACC may explain more than 70% of the variability in speech-reception thresholds: the shorter the latency, the better the SRT. Similarly, shorter and less variable reaction times to speech-relevant ST ripples correspond to a better SRT (Veugen, 2017; Veugen et al., 2022). Thus, a proposed change in the fitting should at least provide a reliable predictor for shorter ripple reaction times (Figure 11) and shorter N1-latencies, both of which can be immediately assessed after the fitting change. Here, we will briefly sketch some possible routes towards this end.

**Rule-based approach.** A first step in the modeling approach is to establish a ‘*rule-based approach*’ on the ‘static’ patient data obtained from the anatomy (experiments 0 and 2), the ECochG result (experiment 1), the SOE measurements (experiment 3), the ECI data (experiment 4) and eCAP (experiment 5). The results of these measurements are assumed to remain constant (although SOE, ECI and eCAP measurements should be repeated at a later stage, for verification) and they are not affected by the fitting. They do, however, provide valuable insights for informed decisions regarding the integrity of the electrode array. For example, it will be important to decide which electrodes to use (activate for hearing), and which ones to remove from the fitting (i.e., inactivate), before optimal fitting parameters of the active CI channels are determined.

In addition, eASSR results (experiment 11) can be used to estimate near-optimal values for the M-levels, which set the range of the pulse-current strengths of the different electrodes (i.e., frequency channels).

**Goal of the ML algorithm.** The ML algorithm will have to learn how to map the current fitting of the (active) array to the reaction-time results of the moving ST ripples, the cortical ASSRs and ACC, and, eventually, the speech-in-noise performance of the individual listener. Clearly, this holy grail poses a daunting challenge, but we are confident that reproducible stimulus–response relationships, together with the idea that these will systematically change in response to changes in the fitting, will provide the necessary information for an ML algorithm to optimize the ultimate goal: a high level of speech-in-noise performance of the CI-user.

Figure 11 illustrates a potential strategy for such an algorithm. Importantly, the algorithm will also have to be fed by prior information regarding the relationship between the spectrotemporal structure of natural human speech and the set of moving spectrotemporal ripples that best cover this structure (e.g., Figure 5). For example, it is known that temporal modulations up to about 10 Hz and spectral modulations below ±1.0 c/o suffice to describe human speech (Chi et al., 1999; Elliott and Theunissen, 2009). Let us call these particular ripples ‘*speech ripples*’. Their contribution to the ST-reaction-time data provides crucial prior knowledge for the algorithm to incorporate in its optimization strategy.

For example, a potential desired outcome of the algorithm for the RT reciprobit lines to speech-relevant ripples (experiment 7) would be to move them towards the median reaction times and slopes that correspond as closely as possible to the distributions obtained from the direct electrical stimulation RT experiments with the CI user (experiments 12 and 13; Figure 10; black line and gray zone in Figure 11). Thus, the difference between the electrical-evoked RT data of the user and the speech-ripple RT data (colored lines) would be expressed as a *quantitative cost* for the algorithm that should be minimized by changing the fitting. Figure 11 illustrates this idea for five different fittings, in which subsequent fitting leads to improved performance, as it gradually reduces the cost.

Similarly, the relation between the eASSRs (experiment 11; Figure 9) from different electrode pairs and acoustically evoked ASSRs for different harmonic complexes (experiment 15) and ACC N1-latencies (experiment 16) can be optimized by assigning a quantitative cost on their differences.

One may expect that when the user can optimally detect the speech ripples, and acoustic ASSRs and short-latency N1-ACCs can be evoked at different frequencies, that speech perception performance of the user, and perhaps even *pitch* perception, will also have improved.

**ML algorithms**. Several possible architectures can be considered to implement the machine-learning algorithm for the data analysis and fitting-to-percept mapping. The algorithm will have to construct a model that embeds the complex relationships between the fitting (input) and the perceptual (RT) and electrophysiological (ASSRs) outcomes for a particular input sound, by incorporating the results from the SOE, ECI, eCAP and direct stimulation experiments (Experiments 1–5, and 11–14). These electrophysiological measurements are considered to reflect the ascending auditory system of the user, whereas the fitting should provide the optimal interface between the acoustic input and the identified auditory system properties. Whether this interface is optimal or not is determined by the acoustic reaction-time and ASSR/ACC experiments (experiments 7 and 15–16), and the eventual speech-recognition scores of experiment 6.

**Figure 12:**
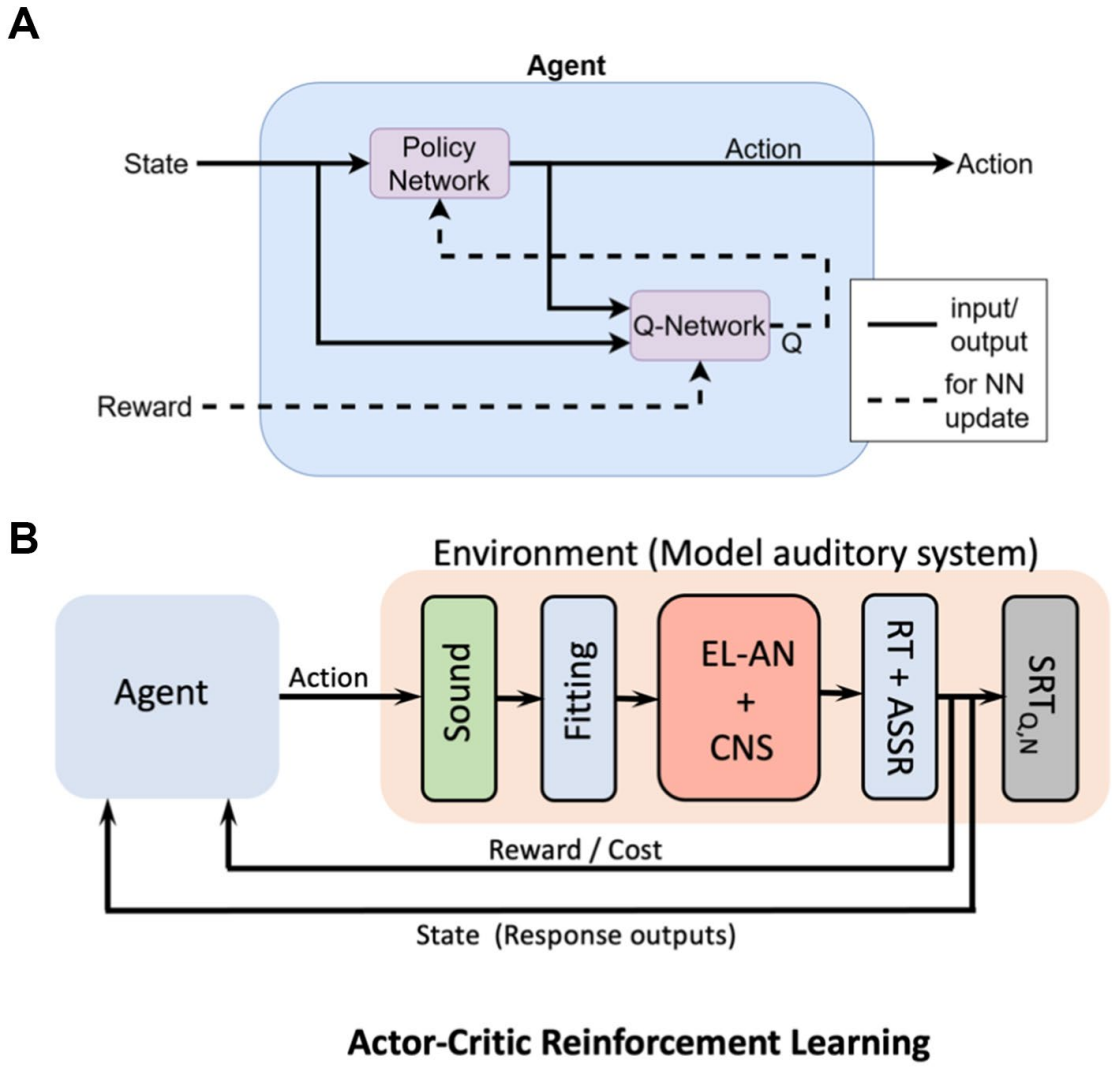
Conceptual machine-learning model for the CI-user’s auditory system, based on the Actor-Critic Reinforcement Learning algorithm. **(A)** The Agent receives two sources of information: the state, consisting of the ‘goal’, the actual output of the system, and the associated cost (reward) for the planned action. The Agent aims to maximize its total reward by training two fully interconnected multilayer neural networks (NN): the Critic (Q-network) which estimates the reward, and the Actor, which programs the Action. **(B)** Conceptual scheme of the total machine-learning system. The Agent’ Action is a proposed change in the Fitting parameters of the speech processor. The Environment contains a model the user’s auditory system, based on the electrophysiological results from all CI participants. The system’s state contains the user’s actual responses to the sound(s), while the ‘goal’ for the Agent is specified by the electrical stimulation RTs and eASSRs (e.g., Figure 11). As the reward increases, the state gradually approaches the goal.

A possible option would be to train a so-called Actor-Critic reinforcement learning algorithm, in which an ‘Agent’ learns how to control the ‘Environment’ (Lee et al., 2016; Haarnoja et al., 2018; Sutton and Barto, 2018). In essence, the Environment is a computational model of the CI-user’s auditory system, which contains all the knowledge constructed from the electrophysiology, anatomy, psychophysics, and direct stimulation results from all users. The control of the Agent is an ‘Action’ that it learns to program through trial and error, and it receives a reward (the inverse of the ‘costs’ described above) for this action, based on how close the action leads to the desired goal (Figure 12B; Haarnoja et al., 2018). The central aim of the Agent is to maximize its total reward. The Agent itself consists of the interaction between two multilayer neural networks, the Q-network (the ‘Critic’) and the Policy, or π-network (the ‘Actor’). The former learns to create an estimate for the reward based on the system’s state, and feeds this estimate to the Actor. The latter learns how to build a ‘better’ action by incorporating the reward and the state. In this way, the Agent gradually learns to control the Environment in an optimal way.

At the present stage, the model in Figure 12 merely serves as a conceptual idea. The exact details of the most appropriate machine-learning algorithm for the problem at hand will need to be worked out and will also heavily depend on the acquired data base (the combined results from the 17 experiments of about 200 CI users) and the built-in prior information. It is conceivable that a different ML architecture might work as good, or even better, as the reinforcement learning algorithm illustrated here (Cassar et al., 2017).

## 4. Discussion

### 4.1. Summary

In this paper, we motivated a comprehensive battery of 17 experimental tests to generate objective, high-quality and reproducible data from all relevant stages in the sensory-neural processing chain of the auditory system of CI users (Table 1). We have argued that this variety of tests is required to provide the essential information of the different processing stages in the system, needed to optimize the personalized fitting of the CI. This includes the quality of the electrode-to-auditory nerve interface, and the integrity of the subsequent ascending auditory pathways, that include binaural integration in case of bilateral implantation. The proposed experiments tease apart the effects of the CI speech processor (which includes the fitting) from the electrode-to-percept pathways (which bypasses the fitting; Figure 2) on the listener’s percepts. Not any single experiment in this list will suffice to reach this goal, as has been amply demonstrated in the literature (Pisoni et al., 2017).

The power of the proposed approach lies in the combined multimodal properties of the data (anatomy, electrophysiology, percept), in which each experimental result can be identified with the different processing stages in the auditory system, taken from a large population of CI-users.

A second important aspect in the approach is the use of abstract stimuli in the experiments, like ST moving ripples, to avoid contamination from cognitive involvement, memory, attentional load, or higher cognitive processing stages that do not relate to the fitting *per se*, but address non-acoustic factors like language skills, familiarity with the sound, etc., (Chi et al., 1999; Elliott and Theunissen, 2009) Moreover, the RT paradigm can be used to immediately assess the effect of a change in the fitting, as an adjustment period will not be required (unlike speech perception). We specifically aim to quantitatively assess the spectral and temporal processing abilities of the user’s auditory system in response to CI stimulation as a proxy for higher level performance, like speech perception in noise.

Thirdly, the use of reaction times to the detection of an abstract stimulus event is a simple task that does not require memory resources or substantial training and can be readily performed by young children and the elderly alike. We have argued (and demonstrated) that the reaction-time paradigm can be reliably used to also assess auditory performance, and that the motor act used to determine the reaction time is not critical (but should be kept constant throughout the tests to enable direct comparisons). In Figure 7B, we illustrated that the task can even be performed by experimental animals and that they can generate detailed information about the listener’s spectral-temporal sensitivity, like the ST modulation transfer function.

To generate the large cumulative data base from many [order 200 (Shafieibavani et al., 2021)] CI-users that could eventually be used by powerful machine-learning algorithms for generating ever-better fitting advises, it will be crucial that the tests are performed in a standardized way across different clinical centers.

It will also be important that the data and algorithms are stored open access and in a standardized way for the benefit of clinicians, hearing-aid manufacturers, researchers, and CI-users throughout the world.

### 4.2. Selected paradigms

In this paper we have focused on a limited set of stimuli (moving ST ripples, LP, HP, or BB noise bursts, pure-tone complexes, tone changes, and simple electrode-stimulation patterns) to characterize and model the ascending auditory system of the CI-user. We discussed several advantages underlying these particular choices, which mainly boil down to their simplicity in terms of parameterization, their possible use for model-based interpretations, and the easy response requirements for the participant by avoiding, or heavily reducing, the cognitive, attentive or memory load.

Clearly, other psychophysical, psychometric, and EEG paradigms and stimuli are possible than those selected here. However, we stress that the main requirement for alternative paradigms will remain that they are easy to perform for the participant, and yield reliable, reproducible, rich, and interpretable results.

### 4.3. Reaction times

Despite the simplicity of the reaction time as a behavioral measure, it accurately reveals the underlying neural processing stages of the sensory-to-motor control loop (Figure 3; Carpenter and Williams, 1995; Glimcher, 2003; Noorani and Carpenter, 2016). Experiments indicate that the reaction time is a highly sensitive measure (one can reliably measure the effect of very subtle stimulus manipulations; Figures 4, 10), is reproducible over extensive time epochs (that may span several months) and contains quantifiable and rich information about the healthy and diseased brain. Moreover, the theoretical model behind the reaction time (LATER) leads to simple, readily quantifiable relationships (straight reciprobit lines) that can be directly interpreted and compared across conditions (and fittings). Moreover, outlier responses can be easily identified and removed from the analysis, if needed.

Note that *absolute* values of RTs are idiosyncratic and method dependent (Noorani and Carpenter, 2016; see, e.g., in Figure 4A the D = 40 ms data for subjects JR, PH, VC). In the LATER model of Figure 3A, such idiosyncratic variability is due to the subject’s prior settings (like experience, expectation, familiarity), response method (eye movement, button press, screen touch), and threshold-decision differences between subjects (like value assignment, risk taking, trigger happiness), as well as the severity of the hearing impairment, but the systematic effects of acoustic manipulations of the stimulus properties (and fitting) within a subject can be well quantified and compared (e.g., Figure 4A, data from PH, and Figure 4B, for different hearing conditions).

Individual differences are not expected to vary between stimulus presentations within an experiment, but care must be taken to prevent unwanted effects like prediction, by fully randomizing trials and stimuli, and by removing potential temporal cues (perceived ‘rhythm’) by introducing a wide-enough range of randomly drawn steady noise periods, D_NOISE_, before the ripple onset. Also, it’s important to be aware about potential stimulus artefacts like the onset of automatic gain control of the CI to the start of the steady noise, which either should be avoided (turn AGC off) or be activated several seconds before the ST ripple.

### 4.4. Limitations

Unfortunately, the proposed methodology is not a panacea for all hearing-impaired. Especially psychometrical experiments, in which participants have to indicate a perceived (small) difference between alternatives or react to near-threshold stimuli (as in audiogram assessments), or perform in reaction-time psychophysics that require many responses, will not be feasible for (very) young children. For these cases, personalized objective data will predominantly be obtained from experiments that do not require active responses, high motivation levels, cognitive skills, and dedicated attention, such as 1–5 (CI-AN interface), 11 and 14, and 15–16 (EEG). By combining these electrophysiological and anatomical data with the more complete data sets from other patients with similar auditory-system and CI characteristics, missing data from participants may be filled in by appropriate estimates extracted from the full data base.

At a later stage (as the child grows up), the personalized data base can be gradually extended to also include reaction-time psychophysics and psychometrics.

The battery of 17 experiments requires substantial total experimental time that involves several recording sessions of up to 2 h each, which is not feasible for all hearing-impaired either. To alleviate this problem, a large part of the psychophysical tests could be performed at home with the use of a dedicated application on a smartphone or tablet, in which the auditory stimuli are presented directly to the CI-processor over Bluetooth. Meanwhile, perceptual responses and reaction times can be recorded by tapping the touch screen. In this way, participants can determine their own preferred pace, experimental duration, and participation frequency without having to visit the clinic. Preliminary tests with a prototype of such an app indicates that the acquired data can have similar reliability as data recorded in a lab environment (Noordanus et al., in prep.). Such a possibility will also have the advantage to accumulate much more high-quality data over time than would ever be possible in a clinical setting.

### 4.5. Challenges

The *main* challenge will be the development of efficient machine-learning models and adequate cost functions that process the vast amounts of multimodal data from all participating CI-users, and learn to combine the results (fitting parameters, anatomy, CI-AN interface data, EEG ASSR/ACC data, psychophysical data, speech performance results in quiet and in noise) from all listeners pooled, to find the best possible fitting advice for any individual listener, and make good predictions for the different experimental outcomes, including speech-reception performance in noise. If the total data set from about 200 CI-users would cover the full range of potential impairments and EL-AN properties, well-informed predictions could be made for new patients, by feeding their electrophysiological data (experiments 1–5, 11–16) into the model.

Although there is still a long way to go, we are confident that a tight collaboration between multiple clinical centers who commit to a joint consensus on strict implementation of the experimental protocols will form an important first step towards these goals.

A second challenge concerns sound localization. Even if binaural fitting requirements are optimally met, the CI-user still misses the essential spectral-shape cues, which in normal-hearing listeners are generated by the pinnae to localize sound-sources in sagittal planes (up-down and front-back). A fundamental problem for the binaural difference cues is that they allow accurate estimation of the azimuth angle in the horizontal plane (left–right), but the true stimulus location remains highly ambiguous since all locations on the so-called ‘cone of confusion’ generate the same ILDs and ITDs (Van Opstal, 2016). In normal hearing, the spectral pinna cues resolve this ambiguity. They also enable the listener to distinguish frontal from rear locations, and to externalize the percept of auditory events in the world, rather than ‘somewhere inside the head’ along the inter-aural axis as with common headphone listening. Clearly, the subtle spectral cues of normal pinnae will not be useful for the CI-processor. Therefore, coarser spectral-encoding strategies need to be implemented to signal sound-source sagittal-plane directions.

It would already help tremendously, if front-back confusions could be avoided, e.g., by emphasizing the source spectrum within the 3–6 kHz range for sounds in the frontal hemifield. The CI-user could learn to use such a simple cue to disambiguate front from back, and perhaps even externalize the percept (Hofman et al., 1998). In addition, locations within the upper- vs. lower hemispheres could be encoded by a relatively simple spectral modulation (like an elevation-dependent gradient in the same 3–6 kHz band), eventually allowing listeners to identify the elevation of a sound source within each of the four sectors of the surrounding sphere (up-down/front-back), and at finer resolution in azimuth. To include such spectral cues in binaural fitting will also require the implementation of an algorithm that estimates source directions from the signals in the CI microphones. Such algorithms are currently not available, and they will have to rely on coarser spectral cues arising from head and shoulders. Clearly, in cluttered acoustic environments the selection and encoding of relevant sound sources will become a serious challenge.

## Data availability statement

The original contributions presented in the study are included in the article/supplementary material, further inquiries can be directed to the corresponding author.

## Ethics statement

The studies involving human participants were reviewed and approved by Ethics Review Board of the Science Faculty. The patients/participants provided their written informed consent to participate in this study.

## Author contributions

AO wrote the paper and prepared the figures. EN performed the experiments, prepared the figures and wrote the paper. All authors contributed to the article and approved the submitted version.

## Funding

This study was funded by the Dutch Science Foundation for Technological Sciences (NWO-TTW, project NeuroCIMT “Otocontrol” nr. 14899; EN), Horizon 2020 ERC advanced grant “Orient” (693400; AJVO), and (NWO-TTW, project “Otocontrol 2.0” nr. 20414; EN).

## Conflict of interest

The authors declare that the research was conducted in the absence of any commercial or financial relationships that could be construed as a potential conflict of interest.

## Publisher’s note

All claims expressed in this article are solely those of the authors and do not necessarily represent those of their affiliated organizations, or those of the publisher, the editors and the reviewers. Any product that may be evaluated in this article, or claim that may be made by its manufacturer, is not guaranteed or endorsed by the publisher.

## Figures and Tables

**Table:** 

ACC	Auditory change complex (evoked potential to a spectral change)
AN	Auditory nerve
ASSR	Auditory steady state response (spectral component in the EEG)
eASSR	Electrically evoked ASSR
BB	Broadband sound
CI	Cochlear Implant
CI-CI	Bilateral CI (left ear, right ear)
CI-HA	Bimodal listening (CI in one ear, hearing aid in right ear)
CNS	Central nervous system
CT	Computer tomography
dB	decibel (SPL: sound-pressure level; dBA: ambient-weighted)
eCAP	electrically-evoked compound action potential (to assess neural health)
EL-AN	Electrode to auditory nerve (signal transfer)
ECI	Electrode cross impedance
ECochG	Electro-Cochleo-Graphy (to assess residual hearing)
EEG	Electro-encephalogram
FAT	Frequency-allocation table (the CI-electrode – sound spectrum mapping)
HA	Hearing aid
HA-HA	Bilateral listening (HA in both ears)
HL	Hearing loss
HP	High-pass filtered sound
IFD	Interaural frequency difference
ITD	Interaural time difference
ILD	Interaural level difference
LATER	Linear approach to threshold with ergodic rate (reaction-time model)
LP	Low-pass filtered sound.
ML	Machine learning
MRI	Magnetic resonance imaging
MTF	Modulation transfer function
NH	Normal hearing
P	Promptness (inverse of reaction time, in s^−1^)
RT	Reaction time button-press timing for detecting a stimulus onset, in sec
RT-eACC	Rection time to an electrically induced ACC
SEO	Spread of excitation of the auditory nerve to single-electrode stimulation.
SRT	Speech Reception Threshold
SRT_N_	SRT in noise
SRT_Q_	SRT in quiet
ST	Spectrotemporal (as in auditory ST sensitivity, or in moving ST ripple stimuli)
WHO	World Health Organization
